# A cross-sectional survey of COVID-19: attitude and prevention practice among Syrians^☆^

**DOI:** 10.1016/j.heliyon.2022.e09124

**Published:** 2022-03-18

**Authors:** Batoul Bakkar, Fatema Mohsen, Humam Armashi, Marah Marrawi, Nizar Aldaher

**Affiliations:** a Faculty of Medicine, Syrian Private University, Damascus, Syria; b Department of Statistics, Syrian Private University, Damascus, Syria; c Professor in Infectious Diseases, Department of Internal Medicine, Faculty of Medicine, Damascus University, Damascus, Syria; d Professor in Infectious Diseases, Department of Internal Medicine, Faculty of Medicine, Syrian Private University, Rif Dimashq, Syria

**Keywords:** Attitude, COVID-19, Practice, Pandemic, Prevention, Syria

## Abstract

**Introduction:**

Coronavirus disease of 2019 has overwhelmed public health systems worldwide and forced governments to impose draconian lockdowns on entire populations. With no vaccine or treatment during the early days of the pandemic, it is of paramount importance to assess the public’s awareness about COVID-19 so that prevention-focused educational campaigns can be sufficiently deployed. This study aimed to gauge the Syrian public’s adherence to infection control measures by assessing attitudes and practices during the pandemic which ravaged an already war-torn Syria.

**Methods:**

The web-based cross-sectional study was conducted in March 2020, nearly 11 years into the Syrian crisis. The survey contained 3 sections: socio-demographic characteristics, attitudes, and practice. Multivariable logistic regression analysis was performed to identify factors associated with good practices and negative attitudes. Data were analyzed using the Statistical Package for Social Sciences version 25.0.

**Results:**

Of the 3586 participants, 68.2% were females, 50.8% were unemployed, and 79.2% were college-educated. Only 1402 (39.1%) participants wore face masks when leaving their homes. Multiple logistic regression analysis revealed that female, age, and residence were factors associated with good practices such as avoiding mass gatherings, wearing face masks, and maintaining a 1-meter interpersonal distance. However, age and occupation were factors associated with negative attitudes towards the closure of universities and schools, travel bans, and quarantines for travellers.

**Conclusion:**

This survey highlights the need to address specific populations using various measures; there should be a specialized method of prevention for each occupation, age group, and place of residence to contain further outbreaks of COVID-19. This can be achieved through targeted awareness campaigns.

## 1 Introduction

Since the first incidence of Coronavirus disease of 2019 (COVID-19) was reported at a local seafood market in Wuhan, China [1], COVID-19 has profoundly impacted societies and economies the world over. The virus has continued to spread resulting in increasing morbidity and mortality, hitting the world’s poorest and most vulnerable populations the hardest [2]. On 30 January 2020, the World Health Organization (WHO) declared for the sixth time that the COVID-19 outbreak is a public health emergency of international concern (PHEIC), prompting the organization to adopt and stipulate drastic global measures to stem the tide of the COVID-19 pandemic [2, 3].

The COVID-19 pandemic has forced governments around the world to impose lockdowns and other restrictive measures in an attempt to reduce infection rates [3]. Mandated preventive health measures include social distancing and isolation, avoiding mixing with vulnerable individuals, wearing masks, and practicing high standards of hygiene. Although these measures restrict personal freedoms, they have generated health benefits at the level of both the individual and society at large [4]. They have lowered contact probabilities with vulnerable populations, especially elderly and the immunocompromised; prevented hospitals from reaching their maximum threshold capacity, flattened the infection curve, and lowered its peaks [4].

The theory of planned behaviour (TPB) is a psychological theory that connects beliefs with behaviours. The theory suggests that the combination of attitude, subjective norms, and perceived behavioural control determine an individual's behavioural intentions. While many studies have been conducted to assess knowledge, attitudes, and practices among various populations around the world during this pandemic, only one has assessed knowledge and awareness among Syrians (conducted, as this study is, by Syrian Private University) [5, 6, 7, 8, 9, 10, 11, 12, 13, 14, 15, 16, 17]. There are no studies regarding the attitudes and practices of Syrians during this pandemic and conflict, and at a time when ambiguity and misinformation are rampant, it is important that we fill this gap to better understand the relationship between beliefs and behaviours among the population.

This study aims to assess attitudes and practices towards COVID-19 among the Syrian population, and, to our knowledge, is the first to do so. The objective of this study is to investigate COVID-19 attitude, prevention practice, and associated demographic characteristics among Syrians. The ultimate goal is to analyse the data in pursuit of the following questions: are there any meaningful correlations between sociodemographic variables, attitudes, and infection control practices? If so, can these insights identify knowledge gaps within the population to be targeted by awareness campaigns?

## 2 COVID-19 in Syria

Syria has endured 10 years of conflict, resulting in the worst refugee crisis since World War II. This pandemic has compounded the situation with new struggles [18]: a study conducted during the pandemic has revealed an alarming 83.4% and 69.6% of Syrians are suffering from depressive and anxiety symptoms [19].

On 22 March 2020, Syria announced its first confirmed COVID-19 case [20]. The numbers of COVID-19 cases and deaths continue to rise in Syria [21]. The Syrian healthcare system has been severely damaged and lacks the capacity to contain such a crisis. The estimated number of intensive care unit (ICU) beds with ventilators is only 325, and the theoretical maximum number of cases that can be adequately treated is barely 6,500 [22]. Once this threshold is exceeded, drastic rationing-of-care decisions must be made. Therefore, cooperation with and response to guidance from the WHO and other organizations are of the utmost importance.

Unprecedented measures have been adopted to control the spread of COVID-19 in Syria including isolation and care of suspected and infected individuals; curfews to limit social contact; partial closure of borders; suspension of public transportation; and closure of mosques, shops, parks, restaurants universities, and schools. The public’s adherence to these control measures, which is largely affected by their attitudes and practices towards COVID-19, is crucial to mitigating the further spread of the pandemic. These challenges along with dense residential areas, the impact of war on education systems, and 83% of the population living under the poverty line make Syria highly vulnerable to a severe outbreak [22, 23].

## 3 Methods

### 3.1 Study design and setting

This web-based cross-sectional survey was conducted over 4 days between 31^st^ March and 4^th^ April 2020. The Arabic-language questionnaire, created using google forms, was posted on various social media platforms including Facebook, Instagram, Telegram, and WhatsApp targeting Syrians from all 14 governorates. The authors were responsible for the survey link distribution. A convenience sampling method was used in the study and was the only feasible approach at the time.

### 3.2 Inclusion and exclusion method

This study was conducted during the first lockdown in Syria. In March 2020, the Syrian government imposed a draconian two-month lockdown [20]. In addition, due to the ongoing conflict, large swaths of territory with considerable populations were no longer under government control. Since physical access to the Syrian population was impossible due to the nationwide quarantine and widespread armed conflict, we had no choice but to collect data via the internet. As a result, the only feasible approach to conducting a study with sufficient reach was via online methods. A 3-point and 4-point Likert scale were used to assess attitudes and practices respectively [24, 25]. Due to the differences in both sections, different Likert scales were adopted. Credible published national data regarding the socio-demographic characteristics of Syrians are not available, nor is there a functional postal service. All the above circumstances precluded the use of better sampling techniques.

### 3.3 Study participants

The minimum sample size calculated was 2401 participants based on a confidence interval of 2, and a confidence level of 95%, for a population of 18,284,423 people using a sample size calculator [26]. The inclusion criteria for this study were that participants must be Syrian residents, over the age of 11 years with no known history of COVID-19 infection. Participation was voluntary and consent to participate in the study was obtained by answering a yes-no question. Participants under the age of 18 and over 11 required informed parental consent and were instructed to supply parent/guardian contact information; the researchers were responsible for contacting the parents/guardians to obtain consent before the child was given access to complete the questionnaire. Participants were informed of the option to opt-out of the survey at any time and were assured of the anonymity and confidentiality of their responses. To avoid non-response bias, the survey was distributed during lockdown when the majority of the population were out of work and at home. In addition, graphics interchange formats (GIFs) and social media posts were adapted to appeal to each social group, and the questions were made short and in the form of multiple choice questions that required no typing. The ability for viewers to comment on the link increased the popularity of the survey. To ensure that participants did not resubmit another response, the survey was programmed to disable the back button on the web-browser to prevent participants from returning to the questionnaire.

### 3.4 Study questionnaire and measures

The questionnaire was designed from several published studies to assess participants’ practices and attitudes towards the COVID-19 pandemic [11, 27]. The questionnaire was translated into Arabic and was reviewed by two dialectologists and two infectious disease specialists. To ascertain the validity, they evaluated whether the questions effectively assessed COVID-19 attitude and practice, and checked for double-barrelled and confusing questions. A pilot study was conducted on 20 individuals to assess relevance, clarity, and the acceptability of the questionnaire [28]. The results of the pilot study were excluded from the sample to avoid bias. Modifications were made based on feedback received to facilitate better comprehension before distributing the final questionnaire to the general population.

The questionnaire contained 3 sections (26 questions):

1. “Socio-demographic information” included 10 items: age, gender, residence, education level, occupation, social status, economic status, smoking, alcohol consumption, and number of household members. These questions were presented as multiple choice questions and fill-in questions for age and number of household members.
2. “Attitude” included 8 items with 3 responses each: *agree*, *disagree*, and *I don’t know.* These questions were presented as multiple choice questions.
3. “Practice” included 8 items with 4 responses each: *always*, *sometimes*, *rarely*, and *never.* These questions were presented as multiple choice questions.

The questionnaire and the answers regarding negative attitudes and good practices are provided in appendix 1.

Reliability analysis was applied to determine the internal consistency of the questionnaire. Internal consistency of its items was measured using Cronbach’s alpha coefficient. The items were considered to represent an acceptable level of internal consistency if the Cronbach’s alpha value was within .50–.70, and good if the value was more than .70 [29, 30, 31]. The Cronbach’s alpha value of the Arabic questionnaire was .53.

### 3.5 Conceptual and operational definitions

In the context of this study *attitude* is a set of emotions and beliefs towards Covid-19, and *practice* is a regular behaviour or method aimed towards mitigating the spread and contraction of COVID-19.

By operational definition, “Covid-19 *attitude”* and “*Covid-19 practice*” are measured using a questionnaire containing 8 questions each.

### 3.6 Ethical approval

Ethical approval was obtained from the Institutional Review Board (IRB) of the Faculty of Medicine, Syrian Private University.

### 3.7 Statistical analysis

For categorical variables, reports were presented as frequencies, percentages and means with standard deviations (SD) for continuous variables. Internal consistency of the questionnaire’s items was measured using Cronbach’s alpha coefficient. The chi-square test was applied to compare attitude and practice questions against socio-demographic variables (age, social status, residence, education level, occupation, economic status, and household members). Binary logistic regression analysis using the socio-demographic variables as independent variables was conducted against attitude (disagree with the travel ban, disagree with quarantine for travellers, and disagree with the closure of universities and schools) and practice (avoiding crowded places, wearing face masks, and leaving over a meter between yourself and people) questions as the outcome variable to identify factors associated with negative attitudes and good practices. Odds ratios and their 95% confidence intervals were used to quantify the associations between socio-demographic variables, attitudes, and practices. Data analysis was conducted with Statistical Package for Social Sciences version 25.0 (SPSS Inc., Chicago, IL, United States). Statistical significance was considered at p-values<0.05.

## 4 Results

### 4.1 Socio-demographics characteristics

**Figure 1:**
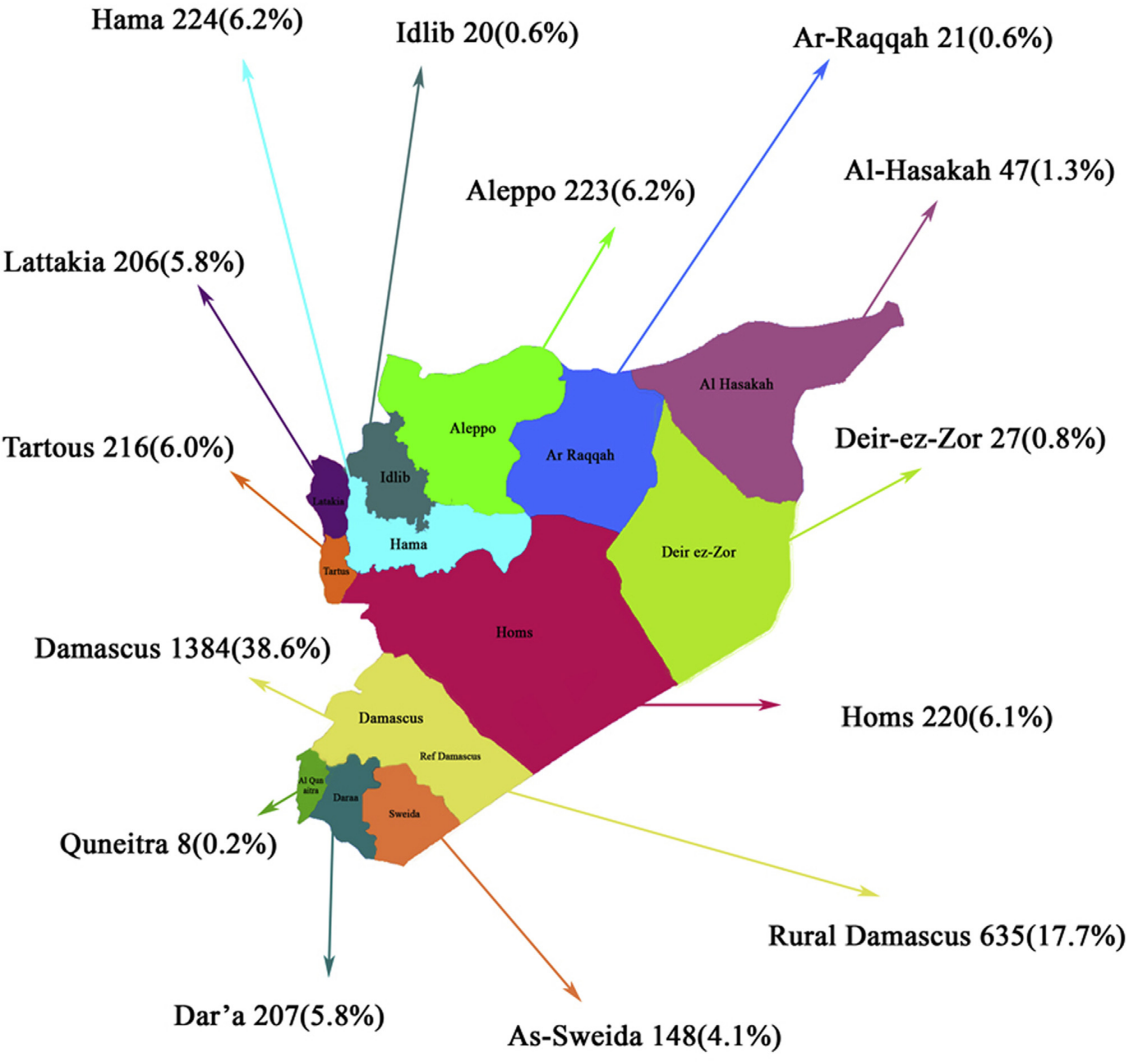
Distribution of participants according to governorates.

Of the 4495 total participants, those who did not meet inclusion criteria were excluded, yielding a final sample of 3586 participants (completion rate = 79.8%). Females accounted for 2444 (68.2%), and males accounted for 1142 (31.8%) of the sample. Participants' ages ranged between 12 and 78 years with the mean being 30 (±10) years. Participants aged 16–30 years were the majority 2789 (77.8%), while participants under 16 years were the minority 59 (1.6%) (Table 1). The majority were single 2279 (63.6%), unemployed 1822 (50.8%), and had attained college/university level education 2839 (79.2%). Smoking and alcohol consumption represented 1064 (29.7%) and 428 (11.9%) respectively. Only 65 (1.8%) knew a COVID-19 infected individual. The majority of participants were residents of Damascus/Rural Damascus 2019 (56.3%) (Figure 1).

**Table 1:** Sociodemographic characteristics of participants: (n = 3586).

Gender (%)	Male	1142 (31.8)
	Female	2444 (68.2)
Age (%)	<16	59 (1.6)
	16–30	2789 (77.8)
	31–45	503 (14.0)
	>45	235 (6.6)
Social Status (%)	Single	2279 (63.5)
	In a relationship	286 (8.0)
	Married	943 (26.3)
	Divorced	46 (1.3)
	Widowed	32 (0.9)
Economic Status (%)	4Excellent	331 (9.2)
	3Good	1761 (49.1)
	2Moderate	1247 (34.8)
	1Poor	247 (6.9)
Education (%)	Primary School	25 (0.7)
	Intermediate School	166 (4.6)
	Secondary school	375 (10.4)
	College/University	2839 (79.2)
	Master’s degree	157 (4.4)
	PhD	24 (0.7)
Occupation (%)	Health care worker	634 (17.7)
	Government institution	283 (7.9)
	Private institution	182 (5.1)
	Business	198 (5.5)
	Military	32 (0.9)
	Unemployed	1822 (50.8)
	Other	435 (12.1)
Household members (%)	0	46 (1.3)
	1–5	2751 (76.7)
	>5	789 (22)

1 Poor: income does not provide essential needs for the family.

2 Moderate: income provides essential needs for the family but no more.

3 Good: income provides essential needs and some luxury requirements.

4 Excellent: income provides luxury requirements.

### 4.2 Practice (infection control) regarding COVID-19

The majority of participants used tissue papers/toilet rolls/handkerchief when sneezing or coughing 3119 (87.0%), avoided public gatherings 2917 (81.3%), abstained from shaking hands and kissing 2755 (76.8%), and washed hands for at least 30 s 2560 (71.4%). Only 213 (5.9%) meet with family members and friends; however, those who wore a face mask, and maintained a 1-meter interpersonal distance when leaving home were only demonstrated by 1402 (39.1%), and 2285 (63.7%) respectively (Table 2).

**Table 2:** Practice (infection control) regarding COVID-19: (n = 3586).

	Always (%)	Sometimes (%)	Rarely (%)	Never (%)
Do you avoid public gatherings (social and religious gatherings)?	2917 (81.3)	465 (13.0)	103 (2.9)	101 (2.8)
Do you still meet with family members and friends?	213 (5.9)	873 (24.4)	1178 (32.8)	1322 (36.9)
Do you wash your hands for at least 30 s?	2560 (71.4)	846 (23.6)	125 (3.5)	55 (1.5)
Do you wear a face facemask when leaving home?	1402 (39.1)	806 (22.5)	457 (12.7)	921 (25.7)
Do you maintain 1-meter between yourself and people when outside?	2285 (63.7)	906 (25.3)	236 (6.6)	159 (4.4)
Have you abstained from shaking hands and kissing?	2755 (76.8)	534 (14.9)	164 (4.6)	133 (3.7)
Do you use a tissue when sneezing or coughing?	3119 (87.0)	343 (9.6)	70 (1.9)	54 (1.5)
Do you refrain from eating takeaway food?	2668 (74.4)	350 (9.8)	243 (6.8)	325 (9.0)

### 4.3 Attitudes regarding (COVID-19)

The majority of participants agreed with the following: infected individuals should be quarantined 3555 (99.1%), universities and schools should be closed 3436 (95.8%), travellers should be quarantined 3362 (93.8%), travel bans between countries 3361 (93.7%), and COVID-19 is a public health concern 3298 (92.0%). Participants agreed that infected individuals have the right to marriage 2350 (65.5%). A minority 741 (20.6%) agreed that lack of faith/religion is the cause of this pandemic (Table 3).

**Table 3:** Attitudes towards COVID-19 crisis: (n = 3586).

	Agree (%)	Disagree (%)	Do Not Know (%)
I believe COVID-19 pandemic is a serious public health issue.	3298 (92.0)	170 (4.7)	118 (3.3)
I believe infected people should be self-isolated.	3555 (99.1)	16 (0.5)	15 (0.4)
I aid the closure of universities, schools. . .	3436 (95.8)	101 (2.8)	49 (1.4)
I aid the travel ban between countries.	3361 (93.7)	202 (5.6)	23 (0.7)
I believe travellers should be quarantined.	3362 (93.8)	172 (4.8)	52 (1.4)
I believe Infected patients have the right to marriage.	2350 (65.5)	191 (5.3)	1045 (29.2)
I believe lack of faith/religion is the cause of this pandemic.	741 (20.6)	2068 (57.7)	777 (21.7)
I believe that an infected individual deserves the infliction of disease.	89 (2.4)	3075 (85.8)	422 (11.8)

### 4.4 Practices and attitudes towards COVID-19 by demographic values

Chi-square test was applied to compare attitude and practice questions against socio-demographic variables. The practice towards avoiding crowded places and mass gatherings significantly differed across gender (χ2 (3, 3586) = 142.6, p < 0.001), age (χ2 (9, 3586) = 32.3, p < 0.001), social status (χ2 (12, 3586) = 36.3, p < 0.001), residence (χ2 (36, 3586) = 76.9, p < 0.001), education (χ2 (15, 3586) = 68.4, p < 0.001), occupation (χ2 (18, 3586) = 78.9, p < 0.001), and economic status (χ2 (9, 3586) = 37.1, p < 0.001). The practice of wearing a face mask when leaving the house significantly differed across gender (χ2 (3, 3586) = 49.1, p < 0.001), age group (χ2 (9, 3586) = 24.4, p = 0.004), area (χ2 (3, 3586) = 12.1, p = 0.007), occupation (χ2 (18, 3586) = 50.6, p < 0.001), and the number of household members (χ2 (6, 3586) = 17.5, p = 0.008) (Table 4).

**Table 4:** Practices of participants by sociodemographic characteristics n (%).

Characteristics		P1-Avoid crowded places and mass gatherings (markets, parties, festivals, and mosques)						P5-Wearing a face mask when leaving the house					
		Always	Rarely	Sometimes	Never	X^2^	P	Never	Rarely	Sometimes	Always	X^2^	P
Gender	Male	44 (3.9)	38 (3.3)	254 (22.2)	806 (70.6)	**142.629**	<0.001	335 (29.3)	172 (15.1)	283 (24.8)	352 (30.8)	**49.055**	<0.001
	Female	57 (2.3)	65 (2.7)	211 (8.6)	2111 (86.4)			586 (24.0)	285 (11.7)	523 (21.4)	1050 (43.0)		
Age group	<16	4 (6.8)	4 (6.8)	5 (8.5)	46 (78.0)	**32.290**	<0.001	17 (28.8)	2 (3.4)	15 (25.4)	25 (42.4)	**24.374**	**0.004**
	16–30	69 (2.5)	72 (2.6)	341 (12.2)	2307 (82.7)			751 (26.9)	361 (12.9)	596 (21.4)	1081 (38.8)		
	31–45	23 (4.6)	16 (3.2)	72 (14.3)	392 (77.9)			114 (22.7)	59 (11.7)	129 (25.6)	201 (40.0)		
	>45	5 (2.1)	11 (4.7)	47 (20.0)	172 (73.2)			39 (16.6)	35 (14.9)	66 (28.1)	95 (40.4)		
Social status	Single	54 (2.4)	56 (2.5)	290 (12.7)	1879 (82.4)	**36.304**	<0.001	614 (26.9)	291 (12.8)	494 (21.7)	880 (38.6)	**17.554**	**0.130**
	Relationship	7 (2.4)	4 (1.4)	33 (11.5)	242 (84.6)			69 (24.1)	45 (15.7)	68 (23.8)	104 (36.4)		
	Married	37 (3.9)	37 (3.9)	125 (13.3)	744 (78.9)			221 (23.4)	113 (12.0)	232 (24.6)	377 (40.0)		
	Divorce	0 (0.0)	4 (8.7)	12 (26.1)	30 (65.2)			8 (17.4)	5 (10.9)	9 (19.6)	24 (52.2)		
	Widow/Widower	3 (9.4)	2 (6.3)	5 (15.6)	22 (68.8)			9 (28.1)	3 (9.4)	3 (9.4)	17 (53.1)		
Residence	Damascus/Rural Damascus	49 (2.4)	59 (2.9)	256 (12.7)	1655 (82.0)	**76.845**	<0.001	497 (24.6)	238 (11.8)	470 (23.3)	814 (40.3)	**47.266**	**0.099**
	Hama	7 (3.1)	4 (1.8)	27 (12.1)	186 (83.0)			67 (29.9)	30 (13.4)	51 (22.8)	76 (33.9)		
	Aleppo	6 (2.7)	3 (1.3)	49 (22.0)	165 (74.0)			70 (31.4)	29 (13.0)	44 (19.7)	80 (35.9)		
	Homs	7 (3.2)	4 (1.8)	23 (10.5)	186 (84.5)			55 (25.0)	32 (14.5)	49 (22.3)	84 (38.2)		
	Tartous	3 (1.4)	8 (3.7)	20 (9.3)	185 (85.6)			45 (20.8)	38 (17.6)	47 (21.8)	86 (39.8)		
	Lattakia	3 (1.5)	4 (1.9)	28 (13.6)	171 (83.0)			59 (28.6)	25 (12.1)	46 (22.3)	76 (36.9)		
	Dar'a	10 (4.8)	11 (5.3)	33 (15.9)	153 (73.9)			59 (28.5)	26 (12.6)	44 (21.3)	78 (37.7)		
	As-Sweida	6 (4.1)	3 (2.0)	12 (8.1)	127 (85.8)			32 (21.6)	20 (13.5)	35 (23.6)	61 (41.2)		
	Al-Hasakah	3 (6.4)	2 (4.3)	7 (14.9)	35 (74.5)			14 (29.8)	8 (17.0)	10 (21.3)	15 (31.9)		
	Deir ez-Zor	3 (11.1)	1 (3.7)	2 (7.4)	21 (77.8)			2 (7.4)	7 (25.9)	4 (14.8)	14 (51.9)		
	Idlib	3 (15.0)	2 (10.0)	5 (25.0)	10 (50.0)			12 (60.0)	1 (5.0)	1 (5.0)	6 (30.0)		
	Ar-Raqqah	1 (4.8)	1 (4.8)	3 (14.3)	16 (76.2)			7 (33.3)	3 (14.3)	3 (14.3)	8 (38.1)		
	Quneitra	0 (0.0)	1 (12.5)	0 (0.0)	7 (87.5)			2 (25.0)	0 (0.0)	2 (25.0)	4 (50.0)		
Areas	Urban	68 (2.8)	69 (2.8)	317 (13.1)	1972 (81.3)	**0.086**	**0.993**	585 (24.1)	310 (12.8)	574 (23.7)	957 (39.4)	**12.114**	**0.007**
	Rural	33 (2.8)	34 (2.9)	148 (12.8)	945 (81.5)			336 (29.0)	147 (12.7)	232 (20.0)	445 (38.4)		
Education	Primary school	3 (12.0)	2 (8.0)	3 (12.0)	17 (68.0)	**68.373**	<0.001	10 (40.0)	4 (16.0)	4 (16.0)	7 (28.0)	19.593	**0.188**
	Secondary school	19 (5.1)	20 (5.3)	56 (14.9)	280 (74.7)			99 (26.4)	48 (12.8)	73 (19.5)	155 (41.3)		
	High school	13 (7.8)	7 (4.2)	16 (9.6)	130 (78.3)			43 (25.9)	10 (6.0)	32 (19.3)	81 (48.8)		
	University/College	63 (2.2)	69 (2.4)	350 (12.3)	2357 (83.0)			725 (25.5)	372 (13.1)	649 (22.9)	1093 (38.5)		
	Master’s degree	2 (1.3)	4 (2.5)	36 (22.9)	115 (73.2)			40 (25.5)	20 (12.7)	41 (26.1)	56 (35.7)		
	PHD	1 (4.2)	1 (4.2)	4 (16.7)	18 (75.0)			4 (16.7)	3 (12.5)	7 (29.2)	10 (41.7)		
Occupation	Health care worker	9 (1.4)	14 (2.2)	91 (14.4)	520 (82.0)	**78.874**	<0.001	130 (20.5)	81 (12.8)	174 (27.4)	249 (39.3)	**50.556**	<0.001
	Government institution	10 (3.5)	10 (3.5)	37 (13.1)	226 (79.9)			67 (23.7)	44 (15.5)	51 (18.0)	121 (42.8)		
	Private institution	5 (2.7)	8 (4.4)	34 (18.7)	135 (74.2)			42 (23.1)	17 (9.3)	61 (33.5)	62 (34.1)		
	Business	7 (3.5)	9 (4.5)	47 (23.7)	135 (68.2)			54 (27.3)	37 (18.7)	41 (20.7)	66 (33.3)		
	Military	4 (12.5)	2 (6.3)	10 (31.3)	16 (50.0)			12 (37.5)	4 (12.5)	8 (25.0)	8 (25.0)		
	Unemployed	48 (2.6)	48 (2.6)	187 (10.3)	1539 (84.5)			504 (27.7)	218 (12.0)	384 (21.1)	716 (39.3)		
	Other	18 (4.1)	12 (2.8)	59 (13.6)	346 (79.5)			112 (25.7)	56 (12.9)	87 (20.0)	180 (41.4)		
Economical status	Excellent	8 (2.4)	8 (2.4)	33 (10.0)	282 (85.2)	**37.094**	<0.001	72 (21.8)	37 (11.2)	71 (21.5)	151 (45.6)	**10.776**	**0.291**
	Good	42 (2.4)	38 (2.2)	224 (12.7)	1457 (82.7)			473 (26.9)	220 (12.5)	395 (22.4)	673 (38.2)		
	Moderate	34 (2.7)	42 (3.4)	170 (13.6)	1001 (80.3)			312 (25.0)	168 (13.5)	292 (23.4)	475 (38.1)		
	Poor	17 (6.9)	15 (6.1)	38 (15.4)	177 (71.7)			64 (25.9)	32 (13.0)	48 (19.4)	103 (41.7)		
Household members	0	2 (4.3)	0 (0.0)	4 (8.7)	40 (87.0)	**3.052**	**0.802**	13 (28.3)	8 (17.4)	12 (26.1)	13 (28.3)	**17.461**	**0.008**
	1–5	75 (2.7)	79 (2.9)	355 (12.9)	2242 (81.5)			675 (24.5)	361 (13.1)	647 (23.5)	1068 (38.8)		
	>5	24 (3.0)	24 (3.0)	106 (13.4)	635 (80.5)			233 (29.5)	88 (11.2)	147 (18.6)	321 (40.7)		

The attitude of participants towards supporting a travel ban between countries significantly differed across gender (χ2 (2, 3586) = 9.4, p = 0.009), education (χ2 (10, 3586) = 33.0, p < 0.001), economic status (χ2 (6, 3586) = 15.9, p = 0.015), and the number of household members (χ2 (4, 3586) = 12.6, p = 0.014). The attitude of participants towards quarantine after travel significantly differed across gender (χ2 (2, 3586) = 25.4, p < 0.001), age (χ2 (6, 3586) = 13.4, p = 0.038), social status (χ2 (8, 3586) = 19.0, p = 0.015), education (χ2 (10, 3586) = 36.0, p < 0.001), and occupation (χ2 (12, 3586) = , p = 0.049) (Table 5).

**Table 5:** Attitudes of participants by sociodemographic characteristics n (%).

Characteristics		A4-I support the travel ban between countries					A5-I believe travellers should be quarantined.				
		Disagree	Agree	IDK	X^2^	P	Disagree	Agree	IDK	X^2^	P
Gender	Male	75 (6.6)	1054 (92.3)	13 (1.1)	9.416	0.009	84 (7.4)	1038 (90.9)	20 (1.8)	25.389	<0.001
	Female	127 (5.2)	2307 (94.4)	10 (0.4)			88 (3.6)	2324 (95.1)	32 (1.3)		
Age group	<16	4 (6.8)	53 (89.8)	2 (3.4)	9.520	0.146	5 (8.5)	52 (88.1)	2 (3.4)	13.350	0.038
	16–30	153 (5.5)	2618 (93.9)	18 (0.6)			146 (5.2)	2603 (93.3)	40 (1.4)		
	31–45	34 (6.8)	467 (92.8)	2 (0.4)			10 (2.0)	486 (96.6)	7 (1.4)		
	>45	11 (4.7)	223 (94.9)	1 (0.4)			11 (4.7)	221 (94.0)	3 (1.3)		
Social status	Single	123 (5.4)	2144 (94.1)	12 (0.5)	6.990	0.538	128 (5.6)	2120 (93.0)	31 (1.4)	18.998	0.015
	Relationship	19 (6.6)	264 (92.3)	3 (1.0)			14 (4.9)	270 (94.4)	2 (0.7)		
	Married	57 (6.0)	879 (93.2)	7 (0.7)			27 (2.9)	899 (95.3)	17 (1.8)		
	Divorce	1 (2.2)	45 (97.8)	0 (0.0)			2 (4.3)	44 (95.7)	0 (0.0)		
	Widow/Widower	2 (6.3)	29 (90.6)	1 (3.1)			1 (3.1)	29 (90.6)	2 (6.3)		
Residence	Damascus/Rural Damascus	113 (5.6)	1895 (93.9)	11 (0.5)	27.550	0.279	103 (5.1)	1889 (93.6)	27 (1.3)	25.451	0.382
	Hama	13 (5.8)	211 (94.2)	0 (0.0)			11 (4.9)	210 (93.8)	3 (1.3)		
	Aleppo	12 (5.4)	208 (93.3)	3 (1.3)			8 (3.6)	209 (93.7)	6 (2.7)		
	Homs	13 (5.9)	205 (93.2)	2 (0.9)			16 (7.3)	202 (91.8)	2 (0.9)		
	Tartous	7 (3.2)	208 (96.3)	1 (0.5)			9 (4.2)	205 (94.9)	2 (0.9)		
	Lattakia	18 (8.7)	185 (89.8)	3 (1.5)			7 (3.4)	196 (95.1)	3 (1.5)		
	Dar'a	7 (3.4)	198 (95.7)	2 (1.0)			5 (2.4)	198 (95.7)	4 (1.9)		
	As-Sweida	8 (5.4)	139 (93.9)	1 (0.7)			7 (4.7)	136 (91.9)	5 (3.4)		
	Al-Hasakah	7 (14.9)	40 (85.1)	0 (0.0)			2 (4.3)	45 (95.7)	0 (0.0)		
	Deir ez-Zor	3 (11.1)	24 (88.9)	0 (0.0)			4 (14.8)	23 (85.2)	0 (0.0)		
	Idlib	0 (0.0)	20 (100.0)	0 (0.0)			0 (0.0)	20 (100.0)	0 (0.0)		
	Ar-Raqqah	0 (0.0)	21 (100.0)	0 (0.0)			0 (0.0)	21 (100.0)	0 (0.0)		
	Quneitra	1 (12.5)	7 (87.5)	0 (0.0)			0 (0.0)	8 (100.0)	0 (0.0)		
Areas	Urban	132 (5.4)	2277 (93.9)	17 (0.7)	0.917	0.632	125 (5.2)	2265 (93.4)	36 (1.5)	2.163	0.339
	Rural	70 (6.0)	1084 (93.4)	6 (0.5)			47 (4.1)	1097 (94.6)	16 (1.4)		
Education	Primary school	3 (12.0)	21 (84.0)	1 (4.0)	32.948	<0.001	3 (12.0)	21 (84.0)	1 (4.0)	35.994	<0.001
	Intermediate school	18 (4.8)	354 (94.4)	3 (0.8)			16 (4.3)	358 (95.5)	1 (0.3)		
	Secondary school	11 (6.6)	150 (90.4)	5 (3.0)			10 (6.0)	146 (88.0)	10 (6.0)		
	University/College	158 (5.6)	2670 (94.0)	11 (0.4)			136 (4.8)	2666 (93.9)	37 (1.3)		
	Master’s degree	9 (5.7)	146 (93.0)	2 (1.3)			7 (4.5)	148 (94.3)	2 (1.3)		
	PHD	3 (12.5)	20 (83.3)	1 (4.2)			0 (0.0)	23 (95.8)	1 (4.2)		
Occupation	Health care worker	34 (5.4)	593 (93.5)	7 (1.1)	18.378	0.105	40 (6.3)	591 (93.2)	3 (0.5)	21.065	0.049
	Government institution	19 (6.7)	263 (92.9)	1 (0.4)			11 (3.9)	271 (95.8)	1 (0.4)		
	Private institution	10 (5.5)	172 (94.5)	0 (0.0)			6 (3.3)	171 (94.0)	5 (2.7)		
	Business	19 (9.6)	178 (89.9)	1 (0.5)			14 (7.1)	180 (90.9)	4 (2.0)		
	Military	2 (6.3)	29 (90.6)	1 (3.1)			1 (3.1)	30 (93.8)	1 (3.1)		
	Unemployed	1035.7)	1710 (93.9)	9 (0.5)			86 (4.7)	1703 (93.5)	33 (1.8)		
	Other	15 (3.4)	416 (95.6)	4 (0.9)			14 (3.2)	416 (95.6)	5 (1.1)		
Economical status	Excellent	21 (8.5)	222 (89.9)	4 (1.6)	15.853	0.015	15 (4.5)	311 (94.0)	5 (1.5)	9.116	0.167
	Good	64 (5.1)	1177 (94.4)	6 (0.5)			87 (4.9)	1656 (94.0)	18 (1.0)		
	Moderate	93 (5.3)	1660 (94.3)	8 (0.5)			56 (4.5)	1170 (93.8)	21 (1.7)		
	Poor	24 (7.3)	302 (91.2)	5 (1.5)			14 (5.7)	225 (91.1)	8 (3.2)		
Household members	0	2 (4.3)	44 (95.7)	0 (0.0)	12.566	0.014	2 (4.3)	43 (93.5)	1 (2.2)	5.759	0.218
	1–5	155 (5.6)	2585 (94.0)	11 (0.4)			125 (4.5)	2592 (94.2)	34 (1.2)		
	>5	45 (5.7)	732 (92.8)	12 (1.5)			45 (5.7)	727 (92.1)	17 (2.2)		

### 4.5 Multiple binary logistic regression analysis

Multiple logistic regression analysis showed that female (vs. male, OR: 1.466, p = 0.013); age group of 16–30 years (OR: 2.726, p = 0.03) and age group of 45 years and above (OR: 10.855, p = 0.008) (vs. 31–45); residence in Hama (OR: 4.306, p = 0.024), Aleppo (OR: 4.680, p = 0.032), Homs (OR: 6.214, p = 0.011), Tartous (OR: 4.590, p = 0.033), Lattakia (OR: 4.194, p = 0.045), and Dar’a (OR: 6.695, p = 0.01) (vs. Damascus/Rural Damascus); economic status of moderate (OR: 1.894, p = 0.005), good (OR: 2.267, p < 0.001), and excellent (OR: 2.070, p = 0.026) (vs. poor) were significantly associated with avoiding crowded places and mass gatherings (Table 6).

**Table 6:** Multiple binary logistic regression analysis on factors significantly associated with practices, and attitudes towards COVID-19.

	p.value	OR	95% C.I.for OR	
			Lower	Lower
**Practice**				
*Avoid crowded places and mass gatherings (markets, parties, festivals, and mosques) (vs not)*				
Female (vs Male)	0.013	1.466	1.085	1.981
Age group 16–30 years (vs 31–45)	0.030	2.726	1.099	6.759
Age group >45 (vs 31–45)	0.008	10.855	1.838	64.086
Residence in Hama (vs Damascus/Rural Damascus)	0.024	4.306	1.207	15.370
Residence in Aleppo (vs Damascus/Rural Damascus)	0.032	4.680	1.143	19.159
Residence in Homs (vs Damascus/Rural Damascus)	0.011	6.214	1.517	25.459
Residence in Tartous (vs Damascus/Rural Damascus)	0.033	4.590	1.128	18.678
Residence in Lattakia (vs Damascus/Rural Damascus)	0.045	4.194	1.030	17.074
Residence in Dar’a (vs Damascus/Rural Damascus)	0.010	6.695	1.577	28.415
Moderate economic status (vs poor)	0.005	1.894	1.216	2.950
Good Economic status (vs poor)	<0.001	2.267	1.448	3.549
Excellent economic status (vs poor)	0.026	2.070	1.090	3.932
*Wearing a face mask when leaving the house (vs not)*				
Female (vs Male)	<0.001	1.455	1.260	1.680
Age group years <16, 31–45, and >45 years (vs 16–30)	<0.001	0.715	0.608	0.841
Occupation in Health Care sector (vs Government, private, Business, Military, Unemployed, and Other)	<0.001	1.394	1.159	1.676
Residence in Damascus/Rural Damascus, Hama, Aleppo, Homs, Tartous, Lattakia, Dar'a, As-Sweida, Deir-ez-Zor, Ar- Raqqah, and Quneitra (vs Al-Hasakah, and Idlib)	<0.001	1.581	1.302	1.920
Urban areas (vs Rural)	0.034	1.166	1.011	1.345
Using only personal toiletries (vs no)	<0.001	0.613	0.525	0.715
*Leaving over a meter between yourself and people when leaving the house*				
Female (vs Male)	<0.001	2.034	1.649	2.508
Age group <16, 31–45, and >45 years (vs 16–30)	<0.001	2.183	1.607	2.964
Primary, secondary, and high school education (vs college/university, master, and PhD)	<0.001	1.728	1.337	2.233
Residence in Damascus/Rural Damascus, Hama, Aleppo, Homs, Tartous, Lattakia, Dar’a, As-Sweida, Deir-ez-Zor, Ar- Raqqah, and Quneitra (vs Al-Hasakah, and Idlib)	<0.001	3.666	2.767	4.857
**Attitude**				
*Disagree with closure of universities, schools (vs. agree)*				
Age group <16 years (vs > 45)	0.006	0.012	0.000	0.272
Age group 16–30 years (vs > 45)	0.001	0.006	0.000	0.122
Age group 31–45 years (vs > 45)	<0.001	0.003	0.000	0.068
Occupation in Business (vs HCW)	0.001	4.379	1.904	10.074
Residence in Damascus/Rural Damascus, Hama, Aleppo, Homs, Tartous, Lattakia, Dar’a, As-Sweida, Deir-ez-Zor, Ar- Raqqah, and Quneitra (vs Al-Hasakah, and Idlib)	0.009∗	3.598	1.383	9.358
Smoking (vs no)	0.006∗	1.905	1.204	3.014
*Disagree with travel ban (vs. agree)*				
Age group 16–30 years (vs 31–45)	<0.001	0.543	0.388	0.759
Age group >45 years (vs 31–45)	0.015	0.435	0.223	0.848
>5, and 1–5 household members (vs 0)	<0.001	0.112	0.082	0.154
Occupation in government, private, business, military, unemployed, and other sectors (vs. HCW)	0.001	0.066	0.014	0.316
Alcohol consumption (vs no)	0.017	0.624	0.424	0.919
*Disagree with quarantine for travellers (vs. agree)*				
Male gender (vs Female)	<0.001	2.043	1.480	2.821
Age group 16–30 years (vs 31–45)	0.027	2.360	1.101	5.057
Occupation in government, private, business, military, unemployed, and other sectors (vs. HCW)	<0.001	0.005	0.001	0.042

Female (vs. male, OR: 1.455, p < 0.001); age groups of <16 years, 31–45 years, and >45 years (vs. 16–30 years, OR: 0.715, p < 0.001); occupation of HCW (vs. government, private, business, military, unemployed, and other sectors, OR: 1.394, p < 0.001); residence in Damascus/Rural Damascus, Hama, Aleppo, Homs, Tartous, Lattakia, Dar’a, As-Sweida, Deir-ez-Zor, Ar- Raqqah, Quneitra (vs. Al-Hasakah, and Idlib, OR: 1.581, p < 0.001); living in urban areas (vs. rural OR: 1.166, p = 0.034); and using only personal toiletries (vs. not OR: 0.613, p < 0.001) were significantly associated with wearing a face mask when leaving the house (Table 6).

Female (vs. male, OR: 2.034, p < 0.001); age groups of <16 years, 31–45 years, >45 years (vs 16–30 years, OR: 2.183, p < 0.001); Primary, secondary, and high school education (vs college/university, master, and PhD, OR:1.728, p < 0.001); residence in Damascus/Rural Damascus, Hama, Aleppo, Homs, Tartous, Lattakia, Dar’a, As-Sweida, Deir-ez-Zor, Ar- Raqqah, and Quneitra (vs Al-Hasakah and Idlib, OR: 3.666, p < 0.001) were significantly associated with maintaining a 1-meter distance from people when outside (Table 6).

Multiple logistic regression analysis showed that age group of <16 years (OR: 0.012, p = 0.006), 16–30 years (OR: 0.006, p = 0.001), 31–45 years (OR: 0.003, p < 0.001) (vs. >45); a career in business (vs HCW, OR: 4.379, p = 0.001); residence in Damascus/Rural Damascus, Damascus, Aleppo, Homs, Tartous, Lattakia, Dar’a, As-Sweida, Deir-ez-Zor, Ar- Raqqah, and Quneitra (vs. Al-Hasakah and Idlib, OR: 3.598, p = 0.009); smoking (vs no, OR: 1.905, p = 0.006) were significantly associated with disagreement regarding the closure of schools and universities (Table 6).

Age group of 16–30 years (OR: 0.543, p < 0.001), >45 years (OR: 0.435, p = 0.015) (vs. 31–45 years); one household member and above (vs. none OR: 0.112, p < 0.001); careers in government, private, business, military, unemployed, and other sectors (vs. HCW, OR: 0.066, p = 0.001); and alcohol consumption (vs. no, OR: 0.624, p = 0.017) were significantly associated with disagreement regarding the travel ban (Table 4).

Male (vs. female, OR: 2.043, p < 0.001); the age group of 16–30 years (vs. 31–45 years, OR: 2.360, p = 0.027); and careers in government, private, business, military, unemployed, and other sectors (vs. HCW, OR: 0.005, p < 0.001) were significantly associated with disagreement regarding quarantining travellers (Table 6).

### 4.6 Study research questions

At the outset of this study, we sought to answer questions about the existence of meaningful correlations between sociodemographic variables, attitudes, and infection control practices; and if such insights could identify knowledge gaps within the population to be targeted by awareness campaigns. By identifying the aforementioned trends, we confirmed both the existence of meaningful associations between certain variables, as well as their utility in future awareness campaigns aimed at improving attitudes and behaviours among the Syrian population.

## 5 Discussion

In the absence of an effective treatment or availability of vaccines against COVID-19 at the time of the survey, the public’s attitude and practice regarding preventive measures towards COVID-19 infection control are crucial to mitigating the spread of the virus. Therefore, it is important to assess the practices and attitudes of the Syrian population; the baseline data can be used by public health policymakers and health professionals to plan effective measures and awareness campaigns targeting specific populations.

At the time of the survey there had only been 10 confirmed cases and 1 death [32]. The majority of Syrian participants exercised caution during the COVID-19 pandemic; 81.3% avoided crowded places and public gatherings, 71.4% washed hands for at least 30 s, and 76.8% abstained from shaking hands and kissing. The figures reported in our study were lower compared with other studies conducted in India, China, and Malaysia [9, 11, 13], but higher than a study conducted in Sudan [15]. WHO and the Centers for Diseases Control and Prevention (CDC) recommended a set of public health interventions (physical distancing, maintaining a distance of two meters between people, avoiding mass gatherings in groups, and other protective measures) to break the transmission cycle of COVID-19 [33, 34]. Results of the present study indicated a lack of adherence by participants towards infection control despite Syrians’ knowledge regarding preventive measures has been shown to be high including avoiding crowded places (99.7%) and washing hands (99.7%) [10].

The present study showed low adherence to wearing face masks similar to a study conducted in Sudan and Egypt [14, 15]. In other studies, conducted in China, Malaysia, Ecuador, and KSA, the number of people who wore face masks was much higher [9, 11, 16, 17]. During the pandemic, the economic status of Syria has deteriorated and significant price increases of personal hygiene items (face masks, hand sanitizers – up to 5,000% increase) have been reported across the country [35]. The drastic increases of both price and demand for masks due to a global shortage of supply is a possible reason behind participants not wearing them [36].

On 12 March 2020, the Syrian government implemented precautionary measures to prevent the spread of the virus, concurrent with the declaration provided by WHO regarding the COVID-19 outbreak evolving into a worldwide pandemic [37]. The vast majority (92.0%) of participants considered the COVID-19 pandemic to be a serious public health issue, much higher than in a Thailand study. Similarly, two other studies in China showed that the majority of people thought that the COVID-19 outbreak was very severe. This attitude may be attributed to the high number of cases and mortality worldwide and the absence of an effective treatment or vaccine at the time of the survey [2, 38].

Shockingly only 65.5% of participants agreed that infected individuals have the right to marry, whereas the rest did not know or disagreed. This kind of stigma reflects negative beliefs and attitudes towards patients with COVID-19. 20.6% of the participants agreed that lack of faith/religion is the cause of this pandemic. A study conducted in Poland revealed that 64.0% of catholic women believed that faith would protect them from COVID-19 and 67.6% declared that faith/spirituality was important for facing the COVID-19 pandemic.

Data revealed that female participants were associated with better practice compared with male participants. A study conducted in China found an association with male gender and hazardous practice [11]. This could be attributed to the fact that a higher proportion of males than females are responsible for providing for their families. As such, they tend to be more preoccupied with work and are less exposed to awareness campaigns on social media, television, and radio. Therefore, the government should target this group for education on preventive control measures to cut the spread of COVID-19.

Multiple logistic regression analysis showed that females; age group of ≥45 years; residence in Hama, Aleppo, Homs, Tartous, Lattakia, and Dar’a (vs Damascus/Rural Damascus); were significantly associated with avoiding crowded places, wearing face masks, and maintaining a 1-meter interpersonal distance. The findings regarding the age group ≥45 years, can be attributed to the participants being more cautious as COVID-19 infection can be severe and lead to death in elderly, chronically ill, and immunodeficient patients. 40.6% and 11,9% of Syrians are hypertensive and diabetic, respectively [37, 39]. This high prevalence of chronic diseases is alarming and underscores the need for targeted awareness campaigns towards younger generations through encouraging the use of face masks and avoiding meeting with older people to protect them from infection.

Multiple logistic regression analysis showed that the age group of 16–30 and occupation in sectors including government, private, business, military, unemployed, and other sectors were significantly associated with negative attitudes towards the travel ban and quarantining travellers. This age group is one of the most economically productive segments of the population; commuting to work and universities requires public and private transportation to be open and unrestricted. Quarantines and measures restricting movement negatively affect those who rely on their daily work to earn a living [35]. Syrians that depend on daily work, such as taxi drivers and small store owners, cannot afford to quarantine, especially after the huge rise in food prices because of war and COVID-19 related factors like panic buying, and reduced store hours [35]. Occupations outside the healthcare system have not received the same education compared with HCW, educating the community about the impact of traveling and not quarantining travellers on the spread of COVID-19 infection is crucial. As this country has no capacity to withstand a pandemic, targeting these groups with awareness campaigns is cost-effective in the long run.

## 6 Limitations

One limitation of this study is that young, well-educated female participants were overrepresented due to the study design; therefore, the results can only be generalized concerning these groups. Another limitation of this study is that participating in the survey required internet access; therefore, the Syrian population had no equal probability to participate in the study. The study included participants from all Syrian governorates, but participants who live in Damascus/Rural Damascus were over-represented in our sample. Credible published national data regarding the socio-demographic characteristics of Syrians are not available to evaluate the representativeness of our sample. The study should indicate that examining the interaction of attitudes and practices was not in the scope of the study though it has been established that attitude affects behaviour [40, 41, 42, 43, 44].

## 7 Conclusion

Our study provides detailed, previously unavailable insight into the attitudes and infection prevention and control practices of the Syrian population, and correlates these with certain socio-demographic variables. The Ministry of Health should be able to leverage this data to develop multimedia awareness campaigns and prevention strategies tailored to each occupation, age group, and locality to eliminate unsafe practices and negative attitudes that contribute to the continued spread of COVID-19. Further research regarding the psychological impacts of the pandemic and acceptability of COVID-19 vaccines is required.

## Declarations

### Author contribution statement

Batoul Bakkar, Fatema Mohsen: Conceived and designed the experiments; Performed the experiments; Wrote the paper.

Humam Armashi: Performed the experiments.

Marah Marrawi: Analyzed ​and interpreted the data.

Nizar Aldaher: Conceived and designed the experiments.

### Funding statement

This research did not receive any specific grant from funding agencies in the public, commercial, or not-for-profit sectors.

### Data availability statement

Data included in article/supplementary ​material/referenced in article.

### Declaration of interests statement

The authors declare no conflict of interest.

### Additional information

No additional information is available for this paper.
