# Supplementary material for: A cross-sectional survey of COVID-19: attitude and prevention practice among Syrians
Source: Heliyon. 2022 Mar 18;8(3):e09124. doi: 10.1016/j.heliyon.2022.e09124 (PMC8933052; doi:10.1016/j.heliyon.2022.e09124)
Supplement: Supplementary_files [file mmc1.docx]

# **Attached Survey:**

## **Appendix 1:**

| **Sociodemographic Characteristics** | |
| --- | --- |
| **Age:**  ⬜<16 ⬜16-30 ⬜31-45 ⬜>45 | **Gender:**  ⬜Male ⬜Female |
| **Marital status:**  ⬜Single ⬜Relationship ⬜Married ⬜Divorced ⬜Widowed | **Educational level:**  ⬜Primary school ⬜Intermediate school ⬜Secondary school ⬜University/College ⬜Master’s Degree ⬜PHD Degree |
| **Occupation:**  ⬜Health care worker ⬜Government institution ⬜Private institution ⬜Business ⬜Military ⬜Unemployed ⬜Other | **Residence:**  ⬜Damascus/Rural Damascus ⬜Hama ⬜Aleppo ⬜Homs ⬜Tartous ⬜Lattakia ⬜Dara’a ⬜As-Sweida ⬜Al Hasakah ⬜Deir-ez-Zor ⬜Idlib ⬜Ar-Raqqah ⬜Quneitra |
| **Area:**  ⬜Rural ⬜Urban | **Economical Status:** ⬜Excellent ⬜Good ⬜Moderate ⬜Poor |
| **Do you smoke?**  ⬜Yes ⬜No | **Do you drink alcohol?** ⬜Yes ⬜No |
| **How many people do you live with?**  ⬜Alone ⬜1-5 ⬜6-10 ⬜11-15 ⬜16-20 ⬜Above 20 | |
|  | |
| **Do you only use your own personal toiletries?**  ⬜Yes ⬜No | |
| **Do you know anyone infected with COVID-19?**  ⬜Yes ⬜No | |

| **Table 2. Practices (Infection Control) towards COVID-19** | | | | |
| --- | --- | --- | --- | --- |
|  | **Always** | **sometimes** | **Rarely** | **Never** |
| **Avoid crowded places and mass gatherings (markets, parties, festivals, and mosques)** | **✓** |  |  |  |
| **Meet friends and relatives nowadays** |  |  |  | **✓** |
| **Refrain from take away food nowadays** | **✓** |  |  |  |
| **Washing hands for at least for 30 seconds** | **✓** |  |  |  |
| **Wearing a face mask when leaving the house** | **✓** |  |  |  |
| **Leaving a distance of over a meter between yourself and people when leaving the house** | **✓** |  |  |  |
| **Abstaining from shaking hands and kissing when greeting people** | **✓** |  |  |  |
| **Using a tissue to cover your mouth and nose when coughing/sneezing.** | **✓** |  |  |  |

| **Table 3. Attitudes towards COVID-19** | | | |
| --- | --- | --- | --- |
|  | **Agree** | **Disagree** | **Do not know** |
| **I believe COVID-19 pandemic is a serious public health issue** |  | **✓** |  |
| **An infected person should be quarantined** |  | **✓** |  |
| **I support the government in the decision to close schools, universities and shops...** |  | **✓** |  |
| **I support the travel ban between countries** |  | **✓** |  |
| **Quarantine for travelers** |  | **✓** |  |
| **COVID-19 treated patients have the right to marriage** |  | **✓** |  |
| **Lack of religion/faith is the cause of this pandemic crisis** | **✓** |  |  |
| **An infected individual deserves this infection** | **✓** |  |  |

# **Statistical Analysis:**

## **Appendix 2:**

| **Characteristics** | | **Attitudes, N(%) or mean (standard deviation)** | | | | | | | | | |
| --- | --- | --- | --- | --- | --- | --- | --- | --- | --- | --- | --- |
|  |  | **A1-I believe COVID-19 pandemic is a serious public health issue** | | | | | **A2-An infected person should be self-isolated** | | | | |
|  |  | **Disagree** | **Agree** | **IDK** | **Chi-Square value** | **P-value** | **Disagree** | **Agree** | **IDK** | **Chi-Square value** | **P-value** |
| **Gender** | **Male** | **70(6.1)** | **1043**  **(91.3)** | **29(2.5)** | **9.766** | **0.008*** | **9(0.8)** | **1129(98.9)** | **4(0.4)** | **4.588** | **0.101** |
|  | **Female** | **100(4.1)** | **2255(92.3)** | **89(3.6)** |  |  | **7(0.3)** | **2426(99.3)** | **11(0.5)** |  |  |
| **Age group** | **<16** | **4(6.8)** | **51(86.4)** | **4(6.8)** | **10.756** | **0.096** | **2(3.4)** | **57(96.6)** | **0(0.0)** | **15.397** | **0.017*** |
|  | **16-30** | **136(4.9)** | **2571(92.2)** | **82(2.9)** |  |  | **13(0.5)** | **2764(99.1)** | **12(0.4)** |  |  |
|  | **31-45** | **21(4.2)** | **456(90.7)** | **26(5.2)** |  |  | **0(0.0)** | **500(99.4)** | **3(0.6)** |  |  |
|  | **>45** | **9(3.8)** | **220(93.6)** | **6(2.6)** |  |  | **1(0.4)** | **234(99.6)** | **0(0.0)** |  |  |
| **Social status** | **Single** | **111(4.9)** | **2100(92.1)** | **68(3.0)** | **4.899** | **0.768** | **13(0.6)** | **2259(99.1)** | **7(0.3)** | **12.416** | **0.134** |
|  | **Relationship** | **10(3.5)** | **267(93.4)** | **9(3.1)** |  |  | **1(0.3)** | **285(99.7)** | **0(0.0)** |  |  |
|  | **Married** | **45(4.8)** | **860(91.2)** | **38(4.0)** |  |  | **2(0.2)** | **934(99.0)** | **7(0.7)** |  |  |
|  | **Divorce** | **3(6.5)** | **42(91.3)** | **1(2.2)** |  |  | **0(0.0)** | **46(100.0)** | **0(0.0)** |  |  |
|  | **Widow/Widower** | **1(3.1)** | **29(90.6)** | **2(6.3)** |  |  | **0(0.0)** | **31(96.9)** | **1(3.1)** |  |  |
| **Residence** | **Damascus/Rural Damascus** | **91(4.5)** | **1866(92.4)** | **62(3.1)** | **42.110** | **0.013*** | **14(0.7)** | **1998(99.0)** | **7(0.3)** | **16.308** | **0.877** |
|  | **Hama** | **19(8.5)** | **198(88.4)** | **7(3.1)** |  |  | **0(0.0)** | **223(99.6)** | **1(0.4)** |  |  |
|  | **Aleppo** | **9(4.0)** | **207(92.8)** | **7(3.1)** |  |  | **1(0.4)** | **222(99.6)** | **0(0.0)** |  |  |
|  | **Homs** | **7(3.2)** | **205(93.2)** | **8(3.6)** |  |  | **0(0.0)** | **218(99.1)** | **2(0.9)** |  |  |
|  | **Tartous** | **11(5.1)** | **199(92.1)** | **6(2.8)** |  |  | **1(0.5)** | **215(99.5)** | **0(0.0)** |  |  |
|  | **Lattakia** | **10(4.9)** | **183(88.8%)** | **13(6.3)** |  |  | **0(0.0)** | **204(99.0)** | **2(1.0)** |  |  |
|  | **Dar’a** | **7(3.4)** | **191(92.3)** | **9(4.3)** |  |  | **0(0.0)** | **206(99.5)** | **1(0.5)** |  |  |
|  | **As-Sweida** | **4(2.7)** | **140(94.6)** | **4(2.7)** |  |  | **0(0.0)** | **146(98.6)** | **2(1.4)** |  |  |
|  | **Al-Hasakah** | **8(17.0)** | **38(80.9)** | **1(2.1)** |  |  | **0(0.0)** | **47(0.0)** | **0(0.0)** |  |  |
|  | **Deir ez-Zor** | **1(3.7)** | **26(96.3)** | **0(0.0)** |  |  | **0(0.0)** | **27(100.0)** | **0(0.0)** |  |  |
|  | **Idlib** | **3(15.0)** | **17(85.0)** | **0(0.0)** |  |  | **0(0.0)** | **20(100.0)** | **0(0.0)** |  |  |
|  | **Ar-Raqqah** | **0(0.0)** | **20(95.2)** | **1(4.8)** |  |  | **0(0.0)** | **21(100.0)** | **0(0.0)** |  |  |
|  | **Quneitra** | **0(0.0)** | **8(100.0)** | **0(0.0)** |  |  | **0(0.0)** | **8(100.0)** | **0(0.0)** |  |  |
| **Areas** | **Urban** | **132(5.4)** | **2215(91.3)** | **79(3.3)** | **8.149** | **0.017*** | **11(0.5)** | **2403(99.1)** | **12(0.5)** | **1.059** | **0.589** |
|  | **Rural** | **38(3.3)** | **1083(93.4)** | **39(3.4)** |  |  | **5(0.4)** | **1152(99.3)** | **3(0.3)** |  |  |
| **Education** | **Primary school** | **0(0.0)** | **21(84.0)** | **4(16.0)** | **39.760** | <0.001 | **2(8.0)** | **23(92.0)** | **0(0.0)** | **54.674** | <0.001 |
|  | **Intermediateschool** | **14(3.7)** | **348(92.8)** | **13(3.5)** |  |  | **3(0.8)** | **370(98.7)** | **2(0.5)** |  |  |
|  | **Secondary school** | **9(5.4)** | **145(87.3)** | **12(7.2)** |  |  | **2(1.2)** | **161(97.0)** | **3(1.8)** |  |  |
|  | **University/College** | **136(4.8)** | **2624(92.4)** | **79(2.8)** |  |  | **8(0.3)** | **2822(99.4)** | **9(0.3)** |  |  |
|  | **Master’s degree** | **10(6.4)** | **141(89.8)** | **6(3.8)** |  |  | **1(0.6)** | **156(99.4)** | **0(0.0)** |  |  |
|  | **PHD** | **1(4.2)** | **19(79.2)** | **4(16.7)** |  |  | **0(0.0)** | **23(95.8)** | **1(4.2)** |  |  |
| **Occupation** | **Health care worker** | **29(4.6)** | **588(92.7)** | **17(2.7)** | **15.554** | **0.213** | **4(0.6)** | **627(98.9)** | **3(0.5)** | **10.616** | **0.562** |
|  | **Government institution** | **14(4.9)** | **260(91.9)** | **9(3.2)** |  |  | **0(0.0)** | **282(99.6)** | **1(0.4)** |  |  |
|  | **Private institution** | **7(3.8)** | **164(90.1)** | **11(6.0)** |  |  | **1(0.5)** | **181(99.5)** | **0(0.0)** |  |  |
|  | **Business** | **14(7.1)** | **176(88.9)** | **8(4.0)** |  |  | **1(0.5)** | **197(99.5)** | **0(0.0)** |  |  |
|  | **Military** | **0(0.0)** | **30(93.8)** | **2(6.3)** |  |  | **0(0.0)** | **31(96.9)** | **1(3.1)** |  |  |
|  | **Unemployed** | **77** | **1686** | **59** |  |  | **9** | **1806** | **7** |  |  |
|  |  | **4.2%** | **92.5%** | **3.2%** |  |  | **0.5%** | **99.1%** | **0.4%** |  |  |
|  | **Other** | **29(6.7)** | **394(90.6)** | **12(2.8)** |  |  | **1(0.2)** | **431(99.1)** | **3(0.7)** |  |  |
| **Economical status** | **Excellent** | **12(3.6)** | **310(93.7)** | **9(2.7)** | **9.374** | **0.154** | **3(0.9)** | **326(98.5)** | **2(0.6)** | **31.571** | <0.001 |
|  | **Good** | **99(5.6)** | **1609(91.4)** | **53(3.0)** |  |  | **3(0.2)** | **1752(99.5)** | **6(0.3)** |  |  |
|  | **Moderate** | **47(3.8)** | **1156(92.7)** | **44(3.5)** |  |  | **4(0.3)** | **1239(99.4)** | **4(0.3)** |  |  |
|  | **Poor** | **12(4.9)** | **223(90.3)** | **12(4.9)** |  |  | **6(2.4)** | **238(96.4)** | **3(1.2)** |  |  |
| **Housemate** | **0** | **0(0.0)** | **44(95.7)** | **2(4.3)** | **3.806** | **0.433** | **0(0.0)** | **46(100.0)** | **0(0.0)** | **3.669** | **0.453** |
|  | **1-5** | **126(4.6)** | **2534(92.1)** | **91(3.3)** |  |  | **10(0.4)** | **2731(99.3)** | **10(0.4)** |  |  |
|  | **>5** | **44(5.6)** | **720(91.3)** | **25(3.2)** |  |  | **6(0.8)** | **778(98.6)** | **5(0.6)** |  |  |

| **Characteristics** | | | **Attitudes, N(%) or mean (standard deviation)** | | | | | | | | | |
| --- | --- | --- | --- | --- | --- | --- | --- | --- | --- | --- | --- | --- |
|  |  |  | **A3-I support the government in the decision to close schools, universities and shops….** | | | | | **A4-I support the travel ban between countries** | | | | |
|  |  |  | **Disagree** | **Agree** | **IDK** | **Chi-Square value** | **P-value** | **Disagree** | **Agree** | **IDK** | **Chi-Square value** | **P-value** |
| **Gender** | **Male** | **N** | **36(3.2)** | **1089(95.4)** | **17(1.5)** | **0.891** | **0.641** | **75(6.6)** | **1054(92.3)** | **13(1.1)** | **9.416** | **0.009*** |
|  | **Female** | **N** | **65(2.7)** | **2347(96.0)** | **32(1.3)** |  |  | **127(5.2)** | **2307(94.4)** | **10(0.4)** |  |  |
| **Age group** | **<16** | **N** | **3(5.1)** | **55(93.2)** | **1(1.7)** | **6.495** | **0.146** | **4(6.8)** | **53(89.8)** | **2(3.4)** | **9.520** | **0.146** |
|  | **16-30** | **N** | **78(2.8)** | **2669(95.7)** | **42(1.5)** |  |  | **153(5.5)** | **2618(93.9)** | **18(0.6)** |  |  |
|  | **31-45** | **N** | **11(2.2)** | **486(96.6)** | **6(1.2)** |  |  | **34(6.8)** | **467(92.8)** | **2(0.4)** |  |  |
|  | **>45** | **N** | **9(3.8)** | **226(96.2)** | **0(0.0)** |  |  | **11(4.7)** | **223(94.9)** | **1(0.4)** |  |  |
| **Social status** | **Single** | **N** | **66(2.9)** | **2181(95.7)** | **32(1.4)** | **5.648** | **0.687** | **123(5.4)** | **2144(94.1)** | **12(0.5)** | **6.990** | **0.538** |
|  | **Relationship** | **N** | **12(4.2)** | **272(95.1)** | **2(0.7)** |  |  | **19(6.6)** | **264(92.3)** | **3(1.0)** |  |  |
|  | **Married** | **N** | **21(2.2)** | **907(96.2)** | **15(1.6)** |  |  | **57(6.0)** | **879(93.2)** | **7(0.7)** |  |  |
|  | **Divorce** | **N** | **1(2.2)** | **45(97.8)** | **0(0.0)** |  |  | **1(2.2)** | **45(97.8)** | **0(0.0)** |  |  |
|  | **Widow/Widower** | **N** | **1(3.1)** | **31(96.9)** | **0(0.0)** |  |  | **2(6.3)** | **29(90.6)** | **1(3.1)** |  |  |
| **Residence** | **Damascus/Rural Damascus** | **N** | **57(2.8)** | **1938(96.0)** | **24(1.2)** | **35.385** | **0.063** | **113(5.6)** | **1895(93.9)** | **11(0.5)** | **27.550** | **0.279** |
|  | **Hama** | **N** | **8(3.6)** | **213(95.1)** | **3(1.3)** |  |  | **13(5.8)** | **211(94.2)** | **0(0.0)** |  |  |
|  | **Aleppo** | **N** | **9(4.0)** | **208(93.3)** | **6(2.7)** |  |  | **12(5.4)** | **208(93.3)** | **3(1.3)** |  |  |
|  | **Homs** | **N** | **3(1.4)** | **216(98.2)** | **1(0.5)** |  |  | **13(5.9)** | **205(93.2)** | **2(0.9)** |  |  |
|  | **Tartous** | **N** | **7(3.2)** | **204(94.4)** | **5(2.3)** |  |  | **7(3.2)** | **208(96.3)** | **1(0.5)** |  |  |
|  | **Lattakia** | **N** | **4(1.9)** | **200(97.1)** | **2(1.0)** |  |  | **18(8.7)** | **185(89.8)** | **3(1.5)** |  |  |
|  | **Dar’a** | **N** | **1(0.5)** | **203(98.1)** | **3(1.4)** |  |  | **7(3.4)** | **198(95.7)** | **2(1.0)** |  |  |
|  | **As-Sweida** | **N** | **4(2.7)** | **141(95.3)** | **3(2.0)** |  |  | **8(5.4)** | **139(93.9)** | **1(0.7)** |  |  |
|  | **Al-Hasakah** | **N** | **3(6.4)** | **43(91.5)** | **1(2.1)** |  |  | **7(14.9)** | **40(85.1)** | **0(0.0)** |  |  |
|  | **Deir ez-Zor** | **N** | **0(0.0)** | **27(100.0)** | **0(0.0)** |  |  | **3(11.1)** | **24(88.9)** | **0(0.0)** |  |  |
|  | **Idlib** | **N** | **2(10.0)** | **17(85.0)** | **1(5.0)** |  |  | **0(0.0)** | **20(100.0)** | **0(0.0)** |  |  |
|  | **Ar-Raqqah** | **N** | **3(14.3)** | **18(85.7)** | **0(0.0)** |  |  | **0(0.0)** | **21(100.0)** | **0(0.0)** |  |  |
|  | **Quneitra** | **N** | **0(0.0)** | **8(100.0)** | **0(0.0)** |  |  | **1(12.5)** | **7(87.5)** | **0(0.0)** |  |  |
| **Areas** | **Urban** | **N** | **72(3.0)** | **2321(95.7)** | **33(1.4)** | **0.628** | **0.730** | **132(5.4)** | **2277(93.9)** | **17(0.7)** | **0.917** | **0.632** |
|  | **Rural** | **N** | **29(2.5)** | **1115(96.1)** | **16(1.4)** |  |  | **70(6.0)** | **1084(93.4)** | **6(0.5)** |  |  |
| **Education** | **Primary school** | **N** | **2(8.0)** | **23(92.0)** | **0(0.0)** | **25.44** | **0.005*** | **3(12.0)** | **21(84.0)** | **1(4.0)** | **32.948** | <0.001 |
|  | **Intermediate school** | **N** | **8(2.1)** | **363(96.8)** | **4(1.1)** |  |  | **18(4.8)** | **354(94.4)** | **3(0.8)** |  |  |
|  | **Secondary school** | **N** | **6(3.6)** | **155(93.4)** | **5(3.0)** |  |  | **11(6.6)** | **150(90.4)** | **5(3.0)** |  |  |
|  | **University/College** | **N** | **73(2.6)** | **2727(96.1)** | **39(1.4)** |  |  | **158(5.6)** | **2670(94.0)** | **11(0.4)** |  |  |
|  | **Master’s degree** | **N** | **12(7.6)** | **145(92.4)** | **0(0.0)** |  |  | **9(5.7)** | **146(93.0)** | **2(1.3)** |  |  |
|  | **PHD** | **N** | **0(0.0)** | **23(95.8)** | **1(4.2)** |  |  | **3(12.5)** | **20(83.3)** | **1(4.2)** |  |  |
| **Occupation** | **Health care worker** | **N** | **13(2.1)** | **616(97.2)** | **5(0.8)** | **22.156** | **0.036*** | **34(5.4)** | **593(93.5)** | **7(1.1)** | **18.378** | **0.105** |
|  | **Government institution** | **N** | **10(3.5)** | **269(95.1)** | **4(1.4)** |  |  | **19(6.7)** | **263(92.9)** | **1(0.4)** |  |  |
|  | **Private institution** | **N** | **3(1.6)** | **175(96.2)** | **4(2.2)** |  |  | **10(5.5)** | **172(94.5)** | **0(0.0)** |  |  |
|  | **Business** | **N** | **14(7.1)** | **179(90.4)** | **5(2.5)** |  |  | **19(9.6)** | **178(89.9)** | **1(0.5)** |  |  |
|  | **Military** | **N** | **1(3.1)** | **31(96.9)** | **0(0.0)** |  |  | **2(6.3)** | **29(90.6)** | **1(3.1)** |  |  |
|  | **Unemployed** | **N** | **51(2.8)** | **1747(95.9)** | **24(1.3)** |  |  | **1035.7)** | **1710(93.9)** | **9(0.5)** |  |  |
|  | **Other** | **N** | **9(2.1)** | **419(96.3)** | **7(1.6)** |  |  | **15(3.4)** | **416(95.6)** | **4(0.9)** |  |  |
| **Economical status** | **Excellent** | **N** | **6(1.8)** | **321(97.0)** | **4(1.2)** | **2.937** | **0.817** | **21(8.5)** | **222(89.9)** | **4(1.6)** | **15.853** | **0.015*** |
|  | **Good** | **N** | **52(3.0)** | **1683(95.6)** | **26(1.5)** |  |  | **64(5.1)** | **1177(94.4)** | **6(0.5)** |  |  |
|  | **Moderate** | **N** | **36(2.9)** | **1197(96.0)** | **14(1.1)** |  |  | **93(5.3)** | **1660(94.3)** | **8(0.5)** |  |  |
|  | **Poor** | **N** | **7(2.8)** | **235(95.1)** | **5(2.0)** |  |  | **24(7.3)** | **302(91.2)** | **5(1.5%)** |  |  |
| **Housemate** | **0** | **N** | **1(2.2)** | **44(95.7)** | **1(2.2)** | **7** | **0.136** | **2(4.3)** | **44(95.7)** | **0(0.0)** | **12.566** | **0.014*** |
|  | **1-5** | **N** | **80(2.9)** | **2641(96.0)** | **30(1.1)** |  |  | **155(5.6)** | **2585(94.0)** | **11(0.4)** |  |  |
|  | **>5** | **N** | **20(2.5)** | **751(95.2)** | **18(2.3)** |  |  | **45(5.7)** | **732(92.8)** | **12(1.5)** |  |  |

| **Characteristics** | | | **Attitudes, N(%) or mean (standard deviation)** | | | | | | | | | |
| --- | --- | --- | --- | --- | --- | --- | --- | --- | --- | --- | --- | --- |
|  |  |  | **A5-**I believe travelers should be quarantined. | | | | | **A6-COVID-19 treated patients have the right to marriage** | | | | |
|  |  |  | **Disagree** | **Agree** | **IDK** | **Chi-Square value** | **P-value** | **Disagree** | **Agree** | **IDK** | **Chi-Square value** | **P-value** |
| **Gender** | **Male** | **N** | **84(7.4)** | **1038(90.9)** | **20(1.8)** | **25.389** | <0.001 | **65(5.7)** | **786(68.8)** | **291(25.5)** | **10.895** | **0.004*** |
|  | **Female** | **N** | **88(3.6)** | **2324(95.1)** | **32(1.3)** |  |  | **126(5.2)** | **1564(64.0)** | **754(30.9)** |  |  |
| **Age group** | **<16** | **N** | **5(8.5)** | **52(88.1)** | **2(3.4)** | **13.350** | **0.038*** | **11(18.6)** | **22(37.3)** | **26(44.1)** | **43.723** | <0.001 |
|  | **16-30** | **N** | **146(5.2)** | **2603(93.3)** | **40(1.4)** |  |  | **157(5.6)** | **1808(64.8)** | **824(29.5)** |  |  |
|  | **31-45** | **N** | **10(2.0)** | **486(96.6)** | **7(1.4)** |  |  | **16(3.2)** | **352(70.0)** | **135(26.8)** |  |  |
|  | **>45** | **N** | **11(4.7)** | **221(94.0)** | **3(1.3)** |  |  | **7(3.0)** | **168(71.5)** | **60(25.5)** |  |  |
| **Social status** | **Single** | **N** | **128(5.6)** | **2120(93.0)** | **31(1.4)** | **18.998** | **0.015*** | **140(6.1)** | **1449(63.6)** | **690(30.3)** | **18.887** | **0.015*** |
|  | **Relationship** | **N** | **14(4.9)** | **270(94.4)** | **2(0.7)** |  |  | **14(4.9)** | **202(70.6)** | **70(24.5)** |  |  |
|  | **Married** | **N** | **27(2.9)** | **899(95.3)** | **17(1.8)** |  |  | **34(3.6)** | **640(67.9)** | **269(28.5)** |  |  |
|  | **Divorce** | **N** | **2(4.3)** | **44(95.7)** | **0(0.0)** |  |  | **1(2.2)** | **35(76.1)** | **10(21.7)** |  |  |
|  | **Widow/Widower** | **N** | **1(3.1)** | **29(90.6)** | **2(6.3)** |  |  | **2(6.3)** | **24(75.0)** | **6(18.8)** |  |  |
| **Residence** | **Damascus/Rural Damascus** | **N** | **103(5.1)** | **1889(93.6)** | **27(1.3)** | **25.451** | **0.382** | **100(5.0)** | **1338(66.3)** | **581(28.8)** | **31.220** | **0.148** |
|  | **Hama** | **N** | **11(4.9)** | **210(93.8)** | **3(1.3)** |  |  | **11(4.9)** | **136(60.7)** | **77(34.4)** |  |  |
|  | **Aleppo** | **N** | **8(3.6)** | **209(93.7)** | **6(2.7)** |  |  | **10(4.5)** | **156(70.0)** | **57(25.6)** |  |  |
|  | **Homs** | **N** | **16(7.3)** | **202(91.8)** | **2(0.9)** |  |  | **18(8.2)** | **144(65.5)** | **58(26.4)** |  |  |
|  | **Tartous** | **N** | **9(4.2)** | **205(94.9)** | **2(0.9)** |  |  | **10(4.6)** | **139(64.4)** | **67(31.0)** |  |  |
|  | **Lattakia** | **N** | **7(3.4)** | **196(95.1)** | **3(1.5)** |  |  | **7(3.4)** | **139(67.5)** | **60(29.1)** |  |  |
|  | **Dar’a** | **N** | **5(2.4)** | **198(95.7)** | **4(1.9)** |  |  | **17(8.2)** | **121(58.5)** | **69(33.3)** |  |  |
|  | **As-Sweida** | **N** | **7(4.7)** | **136(91.9)** | **5(3.4)** |  |  | **14(9.5)** | **90(60.8)** | **44(29.7)** |  |  |
|  | **Al-Hasakah** | **N** | **2(4.3)** | **45(95.7)** | **0(0.0)** |  |  | **4(8.5)** | **32(68.1)** | **11(23.4)** |  |  |
|  | **Deir ez-Zor** | **N** | **4(14.8)** | **23(85.2)** | **0(0.0)** |  |  | **0(0.0)** | **17(63.0)** | **10(37.0)** |  |  |
|  | **Idlib** | **N** | **0(0.0)** | **20(100.0)** | **0(0.0)** |  |  | **0(0.0)** | **15(75.0)** | **5(25.0)** |  |  |
|  | **Ar-Raqqah** | **N** | **0(0.0)** | **21(100.0)** | **0(0.0)** |  |  | **0(0.0)** | **17(81.0)** | **4(19.0)** |  |  |
|  | **Quneitra** | **N** | **0(0.0)** | **8(100.0)** | **0(0.0)** |  |  | **0(0.0)** | **6(75.0)** | **2(25.0)** |  |  |
| **Areas** | **Urban** | **N** | **125(5.2)** | **2265(93.4)** | **36(1.5)** | **2.163** | **0.339** | **122(5.0)** | **1596(65.8)** | **708(29.2)** | **1.324** | **0.516** |
|  | **Rural** | **N** | **47(4.1)** | **1097(94.6)** | **16(1.4)** |  |  | **69(5.9)** | **754(65.0)** | **337(29.1)** |  |  |
| **Education** | **Primary school** | **N** | **3(12.0)** | **21(84.0)** | **1(4.0)** | **35.994** | <0.001 | **1(4.0)** | **14(56.0)** | **10(40.0)** | **47.395** | <0.001 |
|  | **Secondary school** | **N** | **16(4.3)** | **358(95.5)** | **1(0.3)** |  |  | **23(6.1)** | **218(58.1)** | **134(35.7)** |  |  |
|  | **High school** | **N** | **10(6.0)** | **146(88.0)** | **10(6.0)** |  |  | **16(9.6)** | **84(50.6)** | **66(39.8)** |  |  |
|  | **University/College** | **N** | **136(4.8)** | **2666(93.9)** | **37(1.3)** |  |  | **147(5.2)** | **1889(66.5)** | **803(28.3)** |  |  |
|  | **Master’s degree** | **N** | **7(4.5)** | **148(94.3)** | **2(1.3)** |  |  | **4(2.5)** | **125(79.6)** | **28(17.8)** |  |  |
|  | **PHD** | **N** | **0(0.0)** | **23(95.8)** | **1(4.2)** |  |  | **0(0.0)** | **20(83.3)** | **4(16.7)** |  |  |
| **Occupation** | **Health care worker** | **N** | **40(6.3)** | **591(93.2)** | **3(0.5)** | **21.065** | **0.049*** | **30(4.7)** | **460(72.6)** | **144(22.7)** | **38.465** | <0.001 |
|  | **Government institution** | **N** | **11(3.9)** | **271(95.8)** | **1(0.4)** |  |  | **6(2.1)** | **193(68.2)** | **84(29.7)** |  |  |
|  | **Private institution** | **N** | **6(3.3)** | **171(94.0)** | **5(2.7)** |  |  | **8(4.4)** | **133(73.1)** | **41(22.5)** |  |  |
|  | **Business** | **N** | **14(7.1)** | **180(90.9)** | **4(2.0)** |  |  | **9(4.5)** | **128(64.6)** | **61(30.8)** |  |  |
|  | **Military** | **N** | **1(3.1)** | **30(93.8)** | **1(3.1)** |  |  | **2(6.3)** | **16(50.0)** | **14(43.8)** |  |  |
|  | **Unemployed** | **N** | **86(4.7)** | **1703(93.5)** | **33(1.8)** |  |  | **112(6.1)** | **1132(62.1)** | **578(31.7)** |  |  |
|  | **Other** | **N** | **14(3.2)** | **416(95.6)** | **5(1.1)** |  |  | **24(5.5)** | **288(66.2)** | **123(28.3)** |  |  |
| **Economical status** | **Excellent** | **N** | **15(4.5)** | **311(94.0)** | **5(1.5)** | **9.116** | **0.167** | **13(3.9)** | **239(72.2)** | **79(23.9)** | **3.380** | **0.037*** |
|  | **Good** | **N** | **87(4.9)** | **1656(94.0)** | **18(1.0)** |  |  | **97(5.5)** | **1158(65.8)** | **506(28.7)** |  |  |
|  | **Moderate** | **N** | **56(4.5)** | **1170(93.8)** | **21(1.7)** |  |  | **61(4.9)** | **797(63.9)** | **389(31.2)** |  |  |
|  | **Poor** | **N** | **14(5.7)** | **225(91.1)** | **8(3.2)** |  |  | **20(8.1)** | **156(63.2)** | **71(28.7)** |  |  |
| **Housemate** | **0** | **N** | **2(4.3)** | **43(93.5)** | **1(2.2)** | **5.759** | **0.218** | **2(4.3)** | **33(71.7)** | **11(23.9)** | **1.120** | **0.891** |
|  | **1-5** | **N** | **125(4.5)** | **2592(94.2)** | **34(1.2)** |  |  | **145(5.3)** | **1807(65.7)** | **799(29.0)** |  |  |
|  | **>5** | **N** | **45(5.7)** | **727(92.1)** | **17(2.2)** |  |  | **44(5.6)** | **510(64.6)** | **235(29.8)** |  |  |

| **Characteristics** | | | **Attitudes, N(%) or mean (standard deviation)** | | | | | | | | | |
| --- | --- | --- | --- | --- | --- | --- | --- | --- | --- | --- | --- | --- |
|  |  |  | **A7-Lack of religion/faith is the cause of this pandemic crisis** | | | | | **A8-An infected individual deserves this infection** | | | | |
|  |  |  | **Disagree** | **Agree** | **IDK** | **Chi-Square value** | **P-value** | **Disagree** | **Agree** | **IDK** | **Chi-Square value** | **P-value** |
| **Gender** | **Male** | **N** | **669(58.6)** | **243(21.3)** | **230(20.1)** | **2.353** | **0.308** | **937(82.0)** | **49(4.3)** | **156(13.7)** | **29.865** | <0.001 |
|  | **Female** | **N** | **1399(57.2)** | **498(20.4)** | **547(22.4)** |  |  | **2138(87.5)** | **40(1.6)** | **266(10.9)** |  |  |
| **Age group** | **<16** | **N** | **24(40.7)** | **17(28.8)** | **18(30.5)** | **10.005** | **0.124** | **48(81.4)** | **1(1.7)** | **10(16.9)** | **18.459** | **0.005*** |
|  | **16-30** | **N** | **1627(58.3)** | **561(20.1)** | **601(21.5)** |  |  | **2414(86.6)** | **71(2.5)** | **304(10.9)** |  |  |
|  | **31-45** | **N** | **291(57.9)** | **110(21.9)** | **102(20.3)** |  |  | **431(85.7)** | **9(1.8)** | **63(12.5)** |  |  |
|  | **>45** | **N** | **126(53.6)** | **53(22.6)** | **56(23.8)** |  |  | **182(77.4)** | **8(3.4)** | **45(19.1)** |  |  |
| **Social status** | **Single** | **N** | **1361(59.7)** | **428(18.8)** | **490(21.5)** | **33.841** | <0.001 | **1974(86.6)** | **52(2.3)** | **253(11.1)** | **10.883** | **0.208** |
|  | **Relationship** | **N** | **183(64.0)** | **55(19.2)** | **48(16.8)** |  |  | **249(87.1)** | **8(2.8)** | **29(10.1)** |  |  |
|  | **Married** | **N** | **483(51.2)** | **234(24.8)** | **226(24.0)** |  |  | **789(83.7)** | **28(3.0)** | **126(13.4)** |  |  |
|  | **Divorce** | **N** | **26(56.5)** | **13(28.3)** | **7(15.2)** |  |  | **35(76.1)** | **1(2.2)** | **10(21.7)** |  |  |
|  | **Widow/Widower** | **N** | **15(46.9)** | **11(34.4)** | **6(18.8)** |  |  | **28(87.5)** | **0(0.0)** | **4(12.5)** |  |  |
| **Residence** | **Damascus/Rural Damascus** | **N** | **1136(56.3)** | **437(21.6)** | **446(22.1)** | **104.733** | <0.001 | **1744(86.4)** | **40(2.0)** | **235(11.6)** | **55.405** | <0.001 |
|  | **Hama** | **N** | **129(57.6)** | **51(22.8)** | **44(19.6)** |  |  | **191(85.3)** | **3(1.3)** | **30(13.4)** |  |  |
|  | **Aleppo** | **N** | **121(54.3)** | **49(22.0)** | **53(23.8)** |  |  | **201(90.1)** | **1(0.4)** | **21(9.4)** |  |  |
|  | **Homs** | **N** | **145(65.9)** | **38(17.3)** | **37(16.8)** |  |  | **196(89.1)** | **7(3.2)** | **17(7.7)** |  |  |
|  | **Tartous** | **N** | **143(66.2)** | **28(13.0)** | **45(20.8)** |  |  | **174(80.6)** | **12(5.6)** | **30(13.9)** |  |  |
|  | **Lattakia** | **N** | **140(68.0)** | **25(12.1)** | **41(19.9)** |  |  | **177(85.9)** | **6(2.9)** | **23(11.2)** |  |  |
|  | **Dar’a** | **N** | **82(39.6)** | **70(33.8)** | **55(26.6)** |  |  | **167(80.7)** | **5(2.4)** | **35(16.9)** |  |  |
|  | **As-Sweida** | **N** | **115(77.7)** | **11(7.4)** | **22(14.9)** |  |  | **121(81.8)** | **6(4.1)** | **21(14.2)** |  |  |
|  | **Al-Hasakah** | **N** | **27(57.4)** | **11(23.4)** | **9(19.1)** |  |  | **42(89.4)** | **3(6.4)** | **2(4.3)** |  |  |
|  | **Deir ez-Zor** | **N** | **10(37.0)** | **8(29.6)** | **9(33.3)** |  |  | **21(77.8)** | **3(11.1)** | **3(11.1)** |  |  |
|  | **Idlib** | **N** | **7(35.0)** | **5(25.0)** | **8(40.0)** |  |  | **19(95.0)** | **0(0.0)** | **1(5.0)** |  |  |
|  | **Ar-Raqqah** | **N** | **8(38.1)** | **5(23.8)** | **8(38.1)** |  |  | **15(71.4)** | **2(9.5)** | **4(19.0)** |  |  |
|  | **Quneitra** | **N** | **5(62.5)** | **3(37.5)** | **0(0.0)** |  |  | **7(87.5)** | **1(12.5)** | **0(0.0)** |  |  |
| **Areas** | **Urban** | **N** | **1439(59.3)** | **472(19.5)** | **515(21.2)** | **9.490** | **0.009*** | **2101(86.6)** | **47(1.9)** | **278(11.5)** | **10.204** | **0.006*** |
|  | **Rural** | **N** | **629(54.2)** | **269(23.2)** | **262(22.6)** |  |  | **974(84.0)** | **42(3.6)** | **144(12.4)** |  |  |
| **Education** | **Primary school** | **N** | **7(28.0)** | **13(52.0)** | **5(20.0)** | **112.692** | <0.001 | **15(60.0)** | **0(0.0)** | **10(40.0)** | **64.938** | <0.001 |
|  | **Secondary school** | **N** | **170(45.3)** | **115(30.7)** | **90(24.0)** |  |  | **298(79.5)** | **16(4.3)** | **61(16.3)** |  |  |
|  | **High school** | **N** | **54(32.5)** | **60(36.1)** | **52(31.3)** |  |  | **123(74.1)** | **12(7.2)** | **31(18.7)** |  |  |
|  | **University/College** | **N** | **1711(60.3)** | **535(18.8)** | **593(20.9)** |  |  | **2480(87.4)** | **58(2.0)** | **301(10.6)** |  |  |
|  | **Master’s degree** | **N** | **109(69.4)** | **16(10.2)** | **32(20.4)** |  |  | **139(88.5)** | **2(1.3)** | **16(10.2)** |  |  |
|  | **PHD** | **N** | **17(70.8)** | **2(8.3)** | **5(20.8)** |  |  | **20(83.3)** | **1(4.2)** | **3(12.5)** |  |  |
| **Occupation** | **Health care worker** | **N** | **444(70.0)** | **80(12.6)** | **110(17.4)** | **87.128** | <0.001 | **573(90.4)** | **12(1.9)** | **49(7.7)** | **70.855** | <0.001 |
|  | **Government institution** | **N** | **172(60.8)** | **56(19.8)** | **55(19.4)** |  |  | **236(83.4)** | **7(2.5)** | **40(14.1)** |  |  |
|  | **Private institution** | **N** | **120(65.9)** | **27(14.8)** | **35(19.2)** |  |  | **161(88.5)** | **3(1.6)** | **18(9.9)** |  |  |
|  | **Business** | **N** | **96(48.5)** | **55(27.8)** | **47(23.7)** |  |  | **150(75.8)** | **13(6.6)** | **35(17.7)** |  |  |
|  | **Military** | **N** | **10(31.3)** | **15(46.9)** | **7(21.9)** |  |  | **19(59.4)** | **5(15.6)** | **8(25.0)** |  |  |
|  | **Unemployed** | **N** | **993(54.5)** | **395(21.7)** | **434(23.8)** |  |  | **1574(86.4)** | **34(1.9)** | **214(11.7)** |  |  |
|  | **Other** | **N** | **233(53.6)** | **113(26.0)** | **89(20.5)** |  |  | **362(83.2)** | **15(3.4)** | **58(13.3)** |  |  |
| **Economical status** | **Excellent** | **N** | **202(61.0)** | **65(19.6)** | **64(19.3)** | **36.406** | <0.001 | **290(87.6)** | **6(1.8)** | **35(10.6)** | **40.850** | <0.001 |
|  | **Good** | **N** | **1077(61.2)** | **316(17.9)** | **368(20.9)** |  |  | **1539(87.4)** | **43(2.4)** | **179(10.2)** |  |  |
|  | **Moderate** | **N** | **673(54.0)** | **284(22.8)** | **290(23.3)** |  |  | **1061(85.1)** | **23(1.8)** | **163(13.1)** |  |  |
|  | **Poor** | **N** | **116(47.0)** | **76(30.8)** | **55(22.3)** |  |  | **185(74.9)** | **17(6.9)** | **45(18.2)** |  |  |
| **Housemate** | **0** | **N** | **27(58.7)** | **10(21.7)** | **9(19.6)** | **44.992** | <0.001 | **40(87.0)** | **1(2.2)** | **5(10.9)** | **3.685** | **0.450** |
|  | **1-5** | **N** | **1667(60.6)** | **520(18.9)** | **564(20.5)** |  |  | **2375(86.3)** | **66(2.4)** | **310(11.3)** |  |  |
|  | **>5** | **N** | **374(47.4)** | **211(26.7)** | **204(25.9)** |  |  | **660(83.7)** | **22(2.8)** | **107(13.6)** |  |  |

| **Characteristics** | | **Practices, n (%) or mean (standard deviation)** | | | | | | | | | | | |
| --- | --- | --- | --- | --- | --- | --- | --- | --- | --- | --- | --- | --- | --- |
|  |  | **P1-Avoid crowded places and mass gatherings (markets, parties, festivals, and mosques)** | | | | | | **P2-Meet friends and relatives nowadays** | | | | | |
|  | | **Always** | **Rarely** | **Sometimes** | **Never** | **Chi- square test** | **P- value** | **Always** | **Rarely** | **Sometimes** | **Never** | **Chi- square test** | **P- value** |
| **Gender** | **Male** | **44(3.9)** | **38(3.3)** | **254(22.2)** | **806(70.6)** | **142.629** | <0.001 | **297(26.0)** | **389(34.1)** | **359(31.4)** | **97(8.5)** | **107.358** | <0.001 |
|  | **Female** | **57(2.3)** | **65(2.7)** | **211(8.6)** | **2111(86.4)** |  |  | **1025(41.9)** | **789(32.3)** | **514(21.0)** | **116(4.7)** |  |  |
| **Age group** | **<16** | **4(6.8)** | **4(6.8)** | **5(8.5)** | **46(78.0)** | **32.290** | <0.001 | **24(40.7)** | **17(28.8)** | **13(22.0)** | **5(8.5)** | **5.593** | **0.780** |
|  | **16-30** | **69(2.5)** | **72(2.6)** | **341(12.2)** | **2307(82.7)** |  |  | **1047(37.5)** | **908(32.6)** | **674(24.2)** | **160(5.7)** |  |  |
|  | **31-45** | **23(4.6)** | **16(3.2)** | **72(14.3)** | **392(77.9)** |  |  | **173(34.4)** | **176(35.0)** | **122(24.3)** | **32(6.4)** |  |  |
|  | **>45** | **5(2.1)** | **11(4.7)** | **47(20.0)** | **172(73.2)** |  |  | **78(33.2)** | **77(32.8)** | **64(27.2)** | **16(6.8)** |  |  |
| **Social status** | **Single** | **54(2.4)** | **56(2.5)** | **290(12.7)** | **1879(82.4)** | **36.304** | <0.001 | **892(39.1)** | **740(32.5)** | **523(22.9)** | **124(5.4)** | **20.743** | **0.054** |
|  | **Relationship** | **7(2.4)** | **4(1.4)** | **33(11.5)** | **242(84.6)** |  |  | **101(35.3)** | **98(34.3)** | **71(24.8)** | **16(5.6)** |  |  |
|  | **Married** | **37(3.9)** | **37(3.9)** | **125(13.3)** | **744(78.9)** |  |  | **304(32.2)** | **316(35.5)** | **255(27.0)** | **68(7.2)** |  |  |
|  | **Divorce** | **0(0.0)** | **4(8.7)** | **12(26.1)** | **30(65.2)** |  |  | **15(32.6)** | **14(30.4)** | **13(28.3)** | **4(8.7)** |  |  |
|  | **Widow/Widower** | **3(9.4)** | **2(6.3)** | **5(15.6)** | **22(68.8)** |  |  | **10(31.3)** | **10(31.3)** | **11(34.4)** | **1(3.1)** |  |  |
| **Residence** | **Damascus/Rural Damascus** | **49(2.4)** | **59(2.9)** | **256(12.7)** | **1655(82.0)** | **76.845** | <0.001 | **822(40.7)** | **632(31.3)** | **455(22.5)** | **110(5.4)** | **89.445** | <0.001 |
|  | **Hama** | **7(3.1)** | **4(1.8)** | **27(12.1)** | **186(83.0)** |  |  | **67(29.9)** | **80(35.7)** | **65(29.0)** | **12(5.4)** |  |  |
|  | **Aleppo** | **6(2.7)** | **3(1.3)** | **49(22.0)** | **165(74.0)** |  |  | **67(30.0)** | **80(35.9)** | **63(28.3)** | **13(5.8)** |  |  |
|  | **Homs** | **7(3.2)** | **4(1.8)** | **23(10.5)** | **186(84.5)** |  |  | **71(32.3)** | **76(34.5)** | **55(25.0)** | **18(8.2)** |  |  |
|  | **Tartous** | **3(1.4)** | **8(3.7)** | **20(9.3)** | **185(85.6)** |  |  | **81(37.5)** | **72(33.3)** | **54(25.0)** | **9(4.2)** |  |  |
|  | **Lattakia** | **3(1.5)** | **4(1.9)** | **28(13.6)** | **171(83.0)** |  |  | **75(36.4)** | **79(38.3)** | **39(18.9)** | **13(6.3)** |  |  |
|  | **Dar’a** | **10(4.8)** | **11(5.3)** | **33(15.9)** | **153(73.9)** |  |  | **49(23.7)** | **66(31.9)** | **69(33.3)** | **23(11.1)** |  |  |
|  | **As-Sweida** | **6(4.1)** | **3(2.0)** | **12(8.1)** | **127(85.8)** |  |  | **59(39.9)** | **54(36.5)** | **28(18.9)** | **7(4.7)** |  |  |
|  | **Al-Hasakah** | **3(6.4)** | **2(4.3)** | **7(14.9)** | **35(74.5)** |  |  | **15(31.9)** | **15(31.9)** | **14(29.8)** | **3(6.4)** |  |  |
|  | **Deir ez-Zor** | **3(11.1)** | **1(3.7)** | **2(7.4)** | **21(77.8)** |  |  | **10(37.0)** | **7(25.9)** | **9(33.3)** | **1(3.7)** |  |  |
|  | **Idlib** | **3(15.0)** | **2(10.0)** | **5(25.0)** | **10(50.0)** |  |  | **0(0.0)** | **7(35.0)** | **12(60.0)** | **1(5.0)** |  |  |
|  | **Ar-Raqqah** | **1(4.8)** | **1(4.8)** | **3(14.3)** | **16(76.2)** |  |  | **3(14.3)** | **7(33.3)** | **9(42.9)** | **2(9.5)** |  |  |
|  | **Quneitra** | **0(0.0)** | **1(12.5)** | **0(0.0)** | **7(87.5)** |  |  | **3(37.5)** | **3(37.5)** | **1(12.5)** | **1(12.5)** |  |  |
| **Areas** | **Urban** | **68(2.8)** | **69(2.8)** | **317(13.1)** | **1972(81.3)** | **0.086** | **0.993** | **962(39.7)** | **774(31.9)** | **565(23.3)** | **125(5.2)** | **29.112** | <0.001 |
|  | **Rural** | **33(2.8)** | **34(2.9)** | **148(12.8)** | **945(81.5)** |  |  | **360(31.0)** | **404(34.8)** | **308(26.6)** | **88(7.6)** |  |  |
| **Education** | **Primary school** | **3(12.0)** | **2(8.0)** | **3(12.0)** | **17(68.0)** | **68.373** | <0.001 | **7(28.0)** | **10(40.0)** | **5(20.0)** | **3(12.0)** | **25.972** | **0.038*** |
|  | **Secondary school** | **19(5.1)** | **20(5.3)** | **56(14.9)** | **280(74.7)** |  |  | **131(34.9)** | **121(32.3)** | **97(25.9)** | **26(6.9)** |  |  |
|  | **High school** | **13(7.8)** | **7(4.2)** | **16(9.6)** | **130(78.3)** |  |  | **57(34.3)** | **45(27.1)** | **51(30.7)** | **13(7.8)** |  |  |
|  | **University/College** | **63(2.2)** | **69(2.4)** | **350(12.3)** | **2357(83.0)** |  |  | **1080(38.0)** | **927(32.7)** | **667(23.5)** | **165(5.8)** |  |  |
|  | **Master’s degree** | **2(1.3)** | **4(2.5)** | **36(22.9)** | **115(73.2)** |  |  | **41(26.1)** | **66(42.0)** | **45(28.7)** | **5(3.2)** |  |  |
|  | **PHD** | **1(4.2)** | **1(4.2)** | **4(16.7)** | **18(75.0)** |  |  | **6(25.0)** | **9(37.5)** | **8(33.3)** | **1(4.2)** |  |  |
| **Occupation** | **Health care worker** | **9(1.4)** | **14(2.2)** | **91(14.4)** | **520(82.0)** | **78.874** | <0.001 | **223(35.2)** | **213(33.6)** | **162(25.6)** | **36(5.7)** | **50.002** | <0.001 |
|  | **Government institution** | **10(3.5)** | **10(3.5)** | **37(13.1)** | **226(79.9)** |  |  | **103(36.4)** | **109(38.5)** | **57(20.1)** | **14(4.9)** |  |  |
|  | **Private institution** | **5(2.7)** | **8(4.4)** | **34(18.7)** | **135(74.2)** |  |  | **57(31.3)** | **70(38.5)** | **44(24.2)** | **11(6.0)** |  |  |
|  | **Business** | **7(3.5)** | **9(4.5)** | **47(23.7)** | **135(68.2)** |  |  | **43(21.7)** | **77(38.9)** | **63(31.8)** | **15(7.6)** |  |  |
|  | **Military** | **4(12.5)** | **2(6.3)** | **10(31.3)** | **16(50.0)** |  |  | **8(25.0)** | **12(37.5)** | **6(18.8)** | **6(18.8)** |  |  |
|  | **Unemployed** | **48(2.6)** | **48(2.6)** | **187(10.3)** | **1539(84.5)** |  |  | **718(39.4)** | **557(30.6)** | **447(24.5)** | **100(5.5)** |  |  |
|  | **Other** | **18(4.1)** | **12(2.8)** | **59(13.6)** | **346(79.5)** |  |  | **170(39.1)** | **140(32.2)** | **94(21.6)** | **31(7.1)** |  |  |
| **Economical status** | **Excellent** | **8(2.4)** | **8(2.4)** | **33(10.0)** | **282(85.2)** | **37.094** | <0.001 | **155(46.8)** | **88(26.6)** | **68(20.5)** | **20(6.0)** | **31.599** | <0.001 |
|  | **Good** | **42(2.4)** | **38(2.2)** | **224(12.7)** | **1457(82.7))** |  |  | **675(38.3)** | **562(31.9)** | **434(24.6)** | **90(5.1)** |  |  |
|  | **Moderate** | **34(2.7)** | **42(3.4)** | **170(13.6)** | **1001(80.3)** |  |  | **419(33.6)** | **434(34.8)** | **308(24.7)** | **86(6.9)** |  |  |
|  | **Poor** | **17(6.9)** | **15(6.1)** | **38(15.4)** | **177(71.7)** |  |  | **73(29.6)** | **94(38.1)** | **63(25.5)** | **17(6.9)** |  |  |
| **Housemate** | **0** | **2(4.3)** | **0(0.0)** | **4(8.7)** | **40(87.0)** | **3.052** | **0.802** | **19(41.3)** | **13(28.3)** | **10(21.7)** | **4(8.7)** | **10.958** | **0.090** |
|  | **1-5** | **75(2.7)** | **79(2.9)** | **355(12.9)** | **2242(81.5)** |  |  | **1039(37.8)** | **913(33.2)** | **642(23.3)** | **157(5.7)** |  |  |
|  | **>5** | **24(3.0)** | **24(3.0)** | **106(13.4)** | **635(80.5)** |  |  | **264(33.5)** | **252(31.9)** | **221(28.0)** | **52(6.6)** |  |  |

| **Characteristics** | |  | **Practices, n (%) or mean (standard deviation)** | | | | | | | | | | | |
| --- | --- | --- | --- | --- | --- | --- | --- | --- | --- | --- | --- | --- | --- | --- |
|  |  |  | **P3-Refrain from take away food nowadays** | | | | | | **P4-Washing hands for at least for 30 seconds** | | | | | |
|  | |  | **Never** | **Rarely** | **Sometimes** | **Always** | **Chi- square test** | **P- value** | **Never** | **Rarely** | **Sometimes** | **Always** | **Chi- square test** | **P- value** |
| **Gender** | **Male** | **N** | 129(11.3) | 95(8.3) | 121(10.6) | 797(69.8) | **21.086** | <0.001 | 27(2.4) | 61(5.3) | 291(25.5) | 763(66.8) | **31.**  **542** | <0.001 |
|  | **Female** | **N** | 196(8.0) | 148(6.1) | 229(9.4) | 1871(76.6) |  |  | 28(1.1) | 64(2.6) | 555(22.7) | 1797(73.5) |  |  |
| **Age group** | **<16** | **N** | 4(6.8) | 3(5.1) | 12(20.3) | 40(67.8) | **23.976** | **0.004*** | 3(5.1) | 3(5.1) | 12(20.3) | 41(69.5) | **54.182** | <0.001 |
|  | **16-30** | **N** | 241(8.6) | 187(6.7) | 282(10.1) | 2079(74.5) |  |  | 42(1.5) | 113(4.1) | 709(25.4) | 1925(69.0) |  |  |
|  | **31-45** | **N** | 55(10.9) | 44(8.7) | 31(6.2) | 373(74.2) |  |  | 4(0.8) | 7(1.4) | 95(18.9) | 397(78.9) |  |  |
|  | **>45** | **N** | 25(10.6) | 9(3.8) | 25(10.6) | 176(74.9) |  |  | 6(2.6) | 2(0.9) | 30(12.8) | 197(83.8) |  |  |
| **Social status** | **Single** | **N** | 188(8.2) | 161(7.1) | 236(10.4) | 1694(74.3) | **17.066** | **0.147** | 38(1.7) | 96(4.2) | 589(25.8) | 1556(68.3) | **45.124** | <0.001 |
|  | **Relationship** | **N** | 24(.4) | 15(5.2) | 31(10.8) | 216(75.5) |  |  | 4(1.4) | 7(2.4) | 72(25.2) | 203(71.0) |  |  |
|  | **Married** | **N** | 105(11.1) | 61(6.5) | 73(7.7) | 704(74.7) |  |  | 11(1.2) | 19(2.0) | 171(18.1) | 742(78.7) |  |  |
|  | **Divorce** | **N** | 4(8.7) | 3(6.5) | 8(17.4) | 31(67.4) |  |  | 1(2.2) | 1(2.2) | 12(26.1) | 32(69.6) |  |  |
|  | **Widow/Widower** | **N** | 4(12.5) | 3(9.4) | 2(6.3) | 23(71.9) |  |  | 1(3.1) | 2(6.3) | 2(6.3) | 27(84.4) |  |  |
| **Residence** | **Damascus/Rural Damascus** | **N** | 182(9.0) | 160(7.9) | 224(11.1) | 1453(72.0) | **114.661** | <0.001 | 34(1.7) | 66(3.3) | 497(24.6) | 1422(70.4) | **65.575** | **0.002*** |
|  | **Hama** | **N** | 16(7.1) | 10(4.5) | 22(9.8) | 176(78.6) |  |  | 1(0.4) | 10(4.5) | 56(25.0) | 157(70.1) |  |  |
|  | **Aleppo** | **N** | 19(8.5) | 20(9.0) | 41(18.4) | 143(64.1) |  |  | 5(2.5) | 11(4.9) | 52(23.3) | 155(69.5) |  |  |
|  | **Homs** | **N** | 17(7.7) | 14(6.4) | 12(5.5) | 177(80.5) |  |  | 2(0.9) | 8(3.6) | 50(22.7) | 160(72.7) |  |  |
|  | **Tartous** | **N** | 19(8.8) | 6(2.8) | 8(3.7) | 183(84.7) |  |  | 4(1.9) | 8(3.7) | 54(25.0) | 150(69.4) |  |  |
|  | **Lattakia** | **N** | 16(7.8) | 9(4.4) | 15(7.3) | 166(80.6) |  |  | 3(1.5) | 4(1.9) | 39(18.9) | 160(77.7) |  |  |
|  | **Dar’a** | **N** | 23(11.1) | 13(6.3) | 15(7.2) | 156(75.4) |  |  | 2(1.0) | 8(3.9) | 34(16.4) | 163(78.7) |  |  |
|  | **As-Sweida** | **N** | 11(7.4) | 4(2.7) | 3(2.0) | 130(87.8) |  |  | 0(0.0) | 2(1.4) | 33(22.3) | 113(76.4) |  |  |
|  | **Al-Hasakah** | **N** | 7(14.9) | 1(2.1) | 2(4.3) | 37(78.7) |  |  | 0(0.0) | 4(8.5) | 15(31.9) | 28(59.6) |  |  |
|  | **Deir ez-Zor** | **N** | 8(29.6) | 2(7.4) | 2(7.4) | 15(55.6) |  |  | 0(0.0) | 1(3.7) | 7(25.9) | 19(70.4) |  |  |
|  | **Idlib** | **N** | 4(20.0) | 3(15.0) | 5(25.0) | 8(40.0) |  |  | 3(15.0) | 0(0.0) | 3(15.0) | 14(70.0) |  |  |
|  | **Ar-Raqqah** | **N** | 2(9.5) | 1(4.8) | 0(0.0) | 18(85.7) |  |  | 1(4.8) | 3(14.3) | 5(23.8) | 12(57.1) |  |  |
|  | **Quneitra** | **N** | 1(12.5) | 0(0.0) | 1(12.5) | 6(75.0) |  |  | 0(0.0) | 0(0.0) | 1(12.5) | 7(87.5) |  |  |
| **Areas** | **Urban** | **N** | 214(8.8) | 168(6.9) | 260(10.7) | 1784(73.5) | **8.519** | **0.036*** | 41(1.7) | 80(3.3) | 589(24.3) | 1716(70.7) | **3.907** | **0.272** |
|  | **Rural** | **N** | 111(9.6) | 75(6.5) | 90(7.8) | 884(76.2) |  |  | 14(1.2) | 45(3.9) | 257(22.2) | 844(72.8) |  |  |
| **Education** | **Primary school** | **N** | 6(24.0) | 1(4.0) | 3(12.0) | 15(60.0) | 32.194 | **0.006*** | 0(0.0) | 2(20.0) | 5(20.0) | 18(72.0) | **23.475** | **0.075** |
|  | **Secondary school** | **N** | 48(12.8) | 30(8.0) | 32(8.5) | 265(70.7) |  |  | 7(1.9) | 21(5.6) | 78(20.8) | 269(71.7) |  |  |
|  | **High school** | **N** | 19(11.4) | 17(10.2) | 19(11.4) | 111(66.9) |  |  | 6(3.6) | 7(4.2) | 28(16.9) | 125(75.3) |  |  |
|  | **University/College** | **N** | 235(8.3) | 179(6.3) | 270(9.5) | 2155(75.9) |  |  | 41(1.4) | 92(3.2) | 697(24.6) | 2009(70.8) |  |  |
|  | **Master’s degree** | **N** | 15(9.6) | 15(9.6) | 22(14.0) | 105(66.9) |  |  | 1(0.6) | 3(1.9) | 34(21.7) | 119(75.8) |  |  |
|  | **PHD** | **N** | 2(8.3) | 1(4.2) | 4(16.7) | 17(70.8) |  |  | 0(0.0) | 0(0.0) | 4(16.7) | 20(83.3) |  |  |
| **Occupation** | **Health care worker** | **N** | 55(8.7) | 50(7.9) | 82(12.9) | 447(70.5) | **50.372** | <0.001 | 6(0.9) | 16(2.5) | 149(23.5) | 463(73.0) | **31.728** | **0.024*** |
|  | **Government institution** | **N** | 29(10.2) | 17(6.0) | 11(3.9) | 226(79.9) |  |  | 5(1.8) | 6(2.1) | 42(14.8) | 230(81.3) |  |  |
|  | **Private institution** | **N** | 21(11.5) | 19(10.4) | 12(6.6) | 130(71.4) |  |  | 2(1.1) | 4(2.2) | 40(22.0) | 136(74.7) |  |  |
|  | **Business** | **N** | 20(10.1) | 20(10.1) | 25(12.6) | 133(67.2) |  |  | 3(1.5) | 8(4.0) | 42(21.2) | 145(73.2) |  |  |
|  | **Military** | **N** | 8(25.2) | 1(3.1) | 5(15.6) | 18(56.3) |  |  | 2(6.3) | 2(6.3) | 9(28.1) | 19(59.4) |  |  |
|  | **Unemployed** | **N** | 155(8.5) | 107(5.9) | 173(9.5) | 1387(76.1) |  |  | 30(1.6) | 73(4.0) | 450(24.7) | 1269(69.6) |  |  |
|  | **Other** | **N** | 37(8.5) | 29(6.7) | 42(9.7) | 327(75.2) |  |  | 7(1.6) | 16(3.7) | 114(26.2) | 298(68.5) |  |  |
| **Economical status** | **Excellent** | **N** | 29(8.8) | 32(9.7) | 45(13.6) | 225(68.0) | **31.906** | <0.001 | 6(1.8) | 11(3.3) | 70(21.1) | 244(73.7) | **24.859** | **0.003*** |
|  | **Good** | **N** | 145(8.2) | 118(6.7) | 195(11.1) | 1303(74.0) |  |  | 19(1.1) | 70(4.0) | 457(26.0) | 1215(69.0) |  |  |
|  | **Moderate** | **N** | 118(9.5) | 79(6.3) | 95(7.6) | 955(76.6) |  |  | 22(1.8) | 39(3.1) | 277(22.2) | 909(72.9) |  |  |
|  | **Poor** | **N** | 33(13.4) | 14(5.7) | 15(6.1) | 185(74.9) |  |  | 8(3.2) | 5(2) | 42(17) | 192(77.7) |  |  |
| **Housemate** | **0** | **N** | 5(10.9) | 1(2.2) | 7(15.2) | 33(71.7) | **13.230** | **0.040** | 1(2.2) | 1(.2.2) | 11(23.9) | 33(71.7) | **5.018** | **0.542** |
|  | **1-5** | **N** | 244(8.9) | 172(6.3) | 257(9.3) | 2078(75.5) |  |  | 39(1.4) | 89(3.2) | 641(23.3) | 1982(72.0) |  |  |
|  | **>5** | **N** | 76(9.6) | 70(8.9) | 86(10.9) | 557(70.6) |  |  | 15(1.9) | 35(4.4) | 194(24.6) | 545(69.1) |  |  |

| **Characteristics** | |  | **Practices, n (%) or mean (standard deviation)** | | | | | | | | | | | |
| --- | --- | --- | --- | --- | --- | --- | --- | --- | --- | --- | --- | --- | --- | --- |
|  |  |  | **P5-Wearing a face mask when leaving the house** | | | | | | **P6-Leaving a distance of over a meter between yourself and people when leaving the house** | | | | | |
|  | |  | **Never** | **Rarely** | **Sometimes** | **Always** | **Chi- square test** | **P- value** | **Never** | **Rarely** | **Sometimes** | **Always** | **Chi- square test** | **P- value** |
| **Gender** | **Male** | **N** | 335(29.3) | 172(15.1) | 283(24.8) | 352(30.8) | **49.055** | <0.001 | 77(6.7) | 104(9.1) | 376(32.9) | 585(51.2) | **116.346** | <0.001 |
|  | **Female** | **N** | 586(24.0) | 285(11.7) | 523()21.4 | 1050(43.00) |  |  | 82(3.4) | 132(5.4) | 530(21.7) | 1700(69.6) |  |  |
| **Age group** | **<16** | **N** | 17(28.8) | 2(3.4) | 15(25.4) | 25(42.4) | **24.374** | **0.004*** | 6(10.2) | 3(5.1) | 12(20.3) | 38(64.4) | **31.081** | <0.001 |
|  | **16-30** | **N** | 751(26.9) | 361(12.9) | 596(21.4) | 1081(38.8) |  |  | 141(5.1) | 197(7.1) | 714(25.6) | 1737(62.3) |  |  |
|  | **31-45** | **N** | 114(22.7) | 59(11.7) | 129(25.6) | 201(40.0) |  |  | 10(2.0) | 26(5.2) | 117(23.3) | 350(69.6) |  |  |
|  | **>45** | **N** | 39(16.6) | 35(14.9) | 66(28.1) | 95(40.4) |  |  | 2(0.9) | 10(4.3) | 63(26.8) | 160(68.1) |  |  |
| **Social status** | **Single** | **N** | 614(26.9) | 291(12.8) | 494(21.7) | 880(38.6) | **17.554** | **0.130** | 118(5.2) | 144(6.3) | 593(26.0) | 1424(62.5) | **34.815** | **0.001*** |
|  | **Relationship** | **N** | 69(24.1) | 45(15.7) | 68(23.8) | 104(36.4) |  |  | 10(3.5) | 31(10.8) | 85(29.7) | 160(55.9) |  |  |
|  | **Married** | **N** | 221(23.4) | 113(12.0) | 232(24.6) | 377(40.0) |  |  | 30(3.2) | 57(6.2) | 207(22.0) | 649(68.8) |  |  |
|  | **Divorce** | **N** | 8(17.4) | 5(10.9) | 9(19.6) | 24(52.2) |  |  | 0(0.0) | 1(2.2) | 15(32.6) | 30(65.2) |  |  |
|  | **Widow/Widower** | **N** | 9(28.1) | 3(9.4) | 3(9.4) | 17(53.1) |  |  | 1(3.1) | 3(9.4) | 6(18.8) | 22(68.8) |  |  |
| **Residence** | **Damascus/Rural Damascus** | **N** | 497(24.6) | 238(11.8) | 470(23.3) | 814(40.3) | **47.266** | **0.099** | 66(3.3) | 114(5.6) | 516(25.6) | 1323(65.5) | **115.143** | <0.001 |
|  |  | **%** | 24.6% | 11.8% | 23.3% | 40.3% |  |  | 3.3% | 5.6% | 25.6% | 65.5% |  |  |
|  | **Hama** | **N** | 67(29.9) | 30(13.4) | 51(22.8) | 76(33.9) |  |  | 13(5.8) | 20(8.9) | 55(24.6) | 136(60.7) |  |  |
|  | **Aleppo** | **N** | 70(31.4) | 29(13.0) | 44(19.7) | 80(35.9) |  |  | 10(4.5) | 19(8.5) | 74(33.2) | 120(53.8) |  |  |
|  | **Homs** | **N** | 55(25.0) | 32(14.5) | 49(22.3) | 84(38.2) |  |  | 12(5.5) | 14(6.4) | 53(24.1) | 141(64.1) |  |  |
|  | **Tartous** | **N** | 45(20.8) | 38(17.6) | 47(21.8) | 86(39.8) |  |  | 13(6.0) | 13(6.0) | 47(21.8) | 143(66.2) |  |  |
|  | **Lattakia** | **N** | 59(28.6) | 25(12.1) | 46(22.3) | 76(36.9) |  |  | 10(4.9) | 9(4.4) | 40(19.4) | 147(71.4) |  |  |
|  | **Dar’a** | **N** | 59(28.5) | 26(12.6) | 44(21.3) | 78(37.7) |  |  | 15(7.2) | 23(11.1) | 50(24.2) | 119(57.5) |  |  |
|  | **As-Sweida** | **N** | 32(21.6) | 20(13.5) | 35(23.6) | 61(41.2) |  |  | 4(2.7) | 11(7.4) | 32(21.6) | 101(68.2) |  |  |
|  | **Al-Hasakah** | **N** | 14(29.8) | 8(17.0) | 10(21.3) | 15(31.9) |  |  | 4(8.5) | 6(12.8) | 18(38.3) | 19(40.4) |  |  |
|  | **Deir ez-Zor** | **N** | 2(7.4) | 7(25.9) | 4(14.8) | 14(51.9) |  |  | 2(7.4) | 3(11.1) | 6(22.2) | 16(59.3) |  |  |
|  | **Idlib** | **N** | 12(60.0) | 1(5.0) | 1(5.0) | 6(30.0) |  |  | 7(35.0) | 2(10.0) | 4(20.0) | 7(35.0) |  |  |
|  | **Ar-Raqqah** | **N** | 7(33.3) | 3(14.3) | 3(14.3) | 8(38.1) |  |  | 3(14.3) | 2(9.5) | 8(38.1) | 8(35.5) |  |  |
|  | **Quneitra** | **N** | 2(25.0) | 0(0.0) | 2(25.0) | 4(50.0) |  |  | 0(0.0) | 0(.0) | 3(37.5) | 5(62.5) |  |  |
| **Areas** | **Urban** | **N** | 585(24.1) | 310(12.8) | 574(23.7) | 957(39.4) | **12.114** | **0.007*** | 99(4.1) | 148(6.1) | 614(25.3) | 1565(64.5) | **5.480** | **0.140** |
|  | **Rural** | **N** | 336(29.0) | 147(12.7) | 232(20.0) | 445(38.4) |  |  | 60(5.2) | 88(7.6) | 292(25.2) | 720(62.1) |  |  |
| **Education** | **Primary school** | **N** | 10(40.0) | 4(16.0) | 4(16.0) | 7(28.0) | 19.593 | **0.188** | 1(4.0) | 4(16.0) | 3(12.0) | 17(68.0) | **28.340** | **0.020*** |
|  | **Secondary school** | **N** | 99(26.4) | 48(12.8) | 73(19.5) | 155(41.3) |  |  | 20(5.3) | 32(8.5) | 93(24.8) | 230(61.3) |  |  |
|  | **High school** | **N** | 43(25.9) | 10(6.0) | 32(19.3) | 81(48.8) |  |  | 13(7.8) | 9(5.4) | 36(21.7) | 108(65.1) |  |  |
|  | **University/College** | **N** | 725(25.5) | 372(13.1) | 649(22.9) | 1093(38.5) |  |  | 123(4.3) | 172(6.1) | 721(25.4) | 1823(64.2) |  |  |
|  | **Master’s degree** | **N** | 40(25.5) | 20(12.7) | 41(26.1) | 56(35.7) |  |  | 2(1.3) | 18(11.5) | 44(28.0) | 93(59.2) |  |  |
|  | **PHD** | **N** | 4(16.7) | 3(12.5) | 7(29.2) | 10(41.7) |  |  | 0(0.0) | 1(4.2) | 9(37.5) | 14(58.3) |  |  |
| **Occupation** | **Health care worker** | **N** | 130(20.5) | 81(12.8) | 174(27.4) | 249(39.3) | **50.556** | <0.001 | 28(4.4) | 38(6.0) | 170(26.8) | 398(62.8) | **37.203** | **0.005*** |
|  | **Government institution** | **N** | 67(23.7) | 44(15.5) | 51(18.0) | 121(42.8) |  |  | 4(1.4) | 18(6.4) | 72(25.4) | 189(66.8) |  |  |
|  | **Private institution** | **N** | 42(23.1) | 17(9.3) | 61(33.5) | 62(34.1) |  |  | 2(1.1) | 11(6.0) | 53(29.1) | 116(63.7) |  |  |
|  | **Business** | **N** | 54(27.3) | 37(18.7) | 41(20.7) | 66(33.3) |  |  | 7(3.5) | 18(9.1) | 61(30.8) | 112(56.6) |  |  |
|  | **Military** | **N** | 12(37.5) | 4(12.5) | 8(25.0) | 8(25.0) |  |  | 5(15.6) | 4(12.5) | 8(25.0) | 15(46.9) |  |  |
|  | **Unemployed** | **N** | 504(27.7) | 218(12.0) | 384(21.1) | 716(39.3) |  |  | 91(5.0) | 121(6.6) | 425(23.3) | 1185(65.0) |  |  |
|  | **Other** | **N** | 112(25.7) | 56(12.9) | 87(20.0) | 180(41.4) |  |  | 22(5.1) | 26(6.0) | 117(26.9) | 270(62.1) |  |  |
| **Economical status** | **Excellent** | **N** | 72(21.8) | 37(11.2) | 71(21.5) | 151(45.6) | **10.776** | **0.291** | 16(4.8) | 16(4.8) | 72(21.8) | 227(68.6) | **12.512** | **0.186** |
|  | **Good** | **N** | 473(26.9) | 220(12.5) | 395(22.4) | 673(38.2) |  |  | 84(4.8) | 125(7.1) | 451(25.6) | 1101(62.5) |  |  |
|  | **Moderate** | **N** | 312(25.0) | 168(13.5) | 292(23.4) | 475(38.1) |  |  | 43(3.4) | 80(6.4) | 327(26.2) | 797(63.9) |  |  |
|  | **Poor** | **N** | 64(25.9) | 32(13.0) | 48(19.4) | 103(41.7) |  |  | 16(6.5) | 15(6.1) | 56(22.7) | 160(64.8) |  |  |
| **Housemate** | **0** | **N** | 13(28.3) | 8(17.4) | 12(26.1) | 13(28.3) | **17.461** | **0.008*** | 3(6.5) | 1(2.2) | 12(26.1) |  | **21.355** | **0.002*** |
|  | **1-5** | **N** | 675(24.5) | 361(13.1) | 647(23.5) | 1068(38.8) |  |  | 114(4.1) | 167(6.1) | 667(24.2) |  |  |  |
|  | **>5** | **N** | 233(29.5) | 88(11.2) | 147(18.6) | 321(40.7) |  |  | 42(5.3) | 68(8.6) | 227(28.8) |  |  |  |

| **Characteristics** | |  | **Practices, n (%) or mean (standard deviation)** | | | | | | | | | | | |
| --- | --- | --- | --- | --- | --- | --- | --- | --- | --- | --- | --- | --- | --- | --- |
|  |  |  | **P7-Abstaining from shaking hands and kissing when greeting people** | | | | | | **P8-Using a tissue to cover your mouth and nose when coughing/sneezing** | | | | | |
|  | |  | **Never** | **Rarely** | **Sometimes** | **Always** | **Chi- square test** | **P- value** | **Never** | **Rarely** | **Sometimes** | **Always** | **Chi- square test** | **P- value** |
| **Gender** | **Male** | **N** | 58(5.1) | 69(6.0) | 218(19.1) | 797(69.8) | **47.013** | <0.001 | 31(2.7) | 33(2.9) | 157(13.7) | 921(80.6) | **62.170** | <0.001 |
|  | **Female** | **N** | 75(3.1) | 95(3.9) | 316(12.9) | 1958(80.1) |  |  | 23(0.9) | 37(1.5) | 186(7.6) | 2198(89.9) |  |  |
| **Age group** | **<16** | **N** | 5(8.5) | 4(6.8) | 6(10.2) | 44(74.6) | **36.964** | <0.001 | 4(6.8) | 3(5.1) | 9(15.3) | 43(72.9) | **40.595** | <0.001 |
|  | **16-30** | **N** | 106(3.8) | 131(4.7) | 456(16.3) | 2096(75.2) |  |  | 43(1.5) | 60(2.2) | 291(10.4) | 2395(85.9) |  |  |
|  | **31-45** | **N** | 19(3.8) | 25(5.0) | 55(10.9) | 404(80.3) |  |  | 3(0.6) | 4(0.8) | 28(5.6) | 468(93.0) |  |  |
|  | **>45** | **N** | 3(1.3) | 4(1.7) | 17(7.2) | 211(89.8) |  |  | 4(1.7) | 3(1.3) | 15(6.4) | 213(90.6) |  |  |
| **Social status** | **Single** | **N** | 81(3.6) | 99(4.3) | 364(16.0) | 1735(76.1) | **11.717** | **0.469** | 33(1.4) | 51(2.2) | 257(11.3) | 1938(85.0) | **29.163** | **0.004*** |
|  | **Relationship** | **N** | 12(4.2) | 15(5.2) | 42(14.7) | 217(75.9) |  |  | 7(2.4) | 4(1.4) | 22(7.7) | 253(88.5) |  |  |
|  | **Married** | **N** | 37(3.9) | 45(4.8) | 123(13.0) | 738(78.3) |  |  | 13(1.4) | 13(1.4) | 58(6.2) | 859(91.1) |  |  |
|  | **Divorce** | **N** | 2(4.3) | 4(8.7) | 3(6.5) | 37(80.4) |  |  | 0(0.0) | 1(2.2) | 4(8.7) | 41(89.1) |  |  |
|  | **Widow/Widower** | **N** | 1(3.1) | 1(3.1) | 2(6.3) | 28(87.5) |  |  | 1(3.1) | 1(3.1) | 2(6.3) | 28(87.5) |  |  |
| **Residence** | **Damascus/Rural Damascus** | **N** | 64(3.2) | 84(4.2) | 282(14.0) | 1589(78.7) | **173.453** | <0.001 | 24(1.2) | 34(1.7) | 211(10.5) | 1750(86.7) | **94.565** | <0.001 |
|  | **Hama** | **N** | 7(3.1) | 9(4.0) | 40(17.9) | 168(75.0) |  |  | 2(0.9) | 8(3.6) | 17(7.6) | 197(87.9) |  |  |
|  | **Aleppo** | **N** | 5(2.2) | 17(7.6) | 45(20.2) | 156(70.0) |  |  | 3(1.3) | 7(3.1) | 23(10.3) | 190(85.2) |  |  |
|  | **Homs** | **N** | 8(3.6) | 6(2.7) | 29(13.2) | 177(80.5) |  |  | 5(2.3) | 2(0.9) | 18(8.2) | 195(88.6) |  |  |
|  | **Tartous** | **N** | 10(4.6) | 8(3.7) | 15(6.9) | 183(84.7) |  |  | 5(2.3) | 7(3.2) | 17(7.9) | 187(86.6) |  |  |
|  | **Lattakia** | **N** | 9(4.4) | 6(2.9) | 16(7.8) | 175(85.0) |  |  | 4(1.9) | 3(1.5) | 16(7.8) | 183(88.8) |  |  |
|  | **Dar’a** | **N** | 10(4.8) | 15(7.2) | 48(23.2) | 134(64.7) |  |  | 1(0.5) | 2(1.0) | 18(8.7) | 186(89.9) |  |  |
|  | **As-Sweida** | **N** | 1(0.7) | 9(6.1) | 28(18.9) | 110(74.3) |  |  | 3(2.0) | 2(1.4) | 6(4.1) | 137(92.6) |  |  |
|  | **Al-Hasakah** | **N** | 4(8.5) | 4(8.5) | 14(29.8) | 25(53.2) |  |  | 1(2.1) | 1(2.1) | 5(10.6) | 40(85.1) |  |  |
|  | **Deir ez-Zor** | **N** | 5(18.5) | 1(3.7) | 6(22.2) | 15(55.6) |  |  | 1(3.7) | 2(7.4) | 3(11.1) | 21(77.8) |  |  |
|  | **Idlib** | **N** | 7(35.0) | 1(5.0) | 5(25.0) | 7(35.0) |  |  | 4(20.0) | 0(0.0) | 4(20.0) | 12(60.0) |  |  |
|  | **Ar-Raqqah** | **N** | 3(14.3) | 3(14.3) | 5(23.8) | 10(47.6) |  |  | 1(4.8) | 1(4.8) | 5(23.8) | 14(66.7) |  |  |
|  | **Quneitra** | **N** | 0(0.0) | 1(12.5) | 1(12.5) | 6(75.0) |  |  | 0(0.0) | 1(12.5) | 0(0.0) | 7(87.5) |  |  |
| **Areas** | **Urban** | **N** | 79(3.3) | 98(4.0) | 326(13.4) | 1923(79.3) | **25.262** | <0.001 | 33(1.4) | 48(2.0) | 246(10.1) | 2099(86.5) | **3.856** | **0.277** |
|  | **Rural** | **N** | 54(4.7) | 66(5.7) | 208(17.9) | 832(71.7) |  |  | 21(1.8) | 22(1.9) | 97(8.4) | 1020(87.9) |  |  |
| **Education** | **Primary school** | **N** | 2(8.0) | 3(12.0) | 4(16.0) | 16(64.0) | 37.288 | **0.001*** | 1(4.0) | 3(12.0) | 3(12.0) | 18(72.0) | **33.121** | **0.005*** |
|  | **Secondary school** | **N** | 22(5.9) | 25(6.7) | 63(16.8) | 265(70.7) |  |  | 5(1.3) | 9(2.4) | 34(9.1) | 327(87.2) |  |  |
|  | **High school** | **N** | 12(7.2) | 15(9.0) | 21(12.7) | 118(71.1) |  |  | 8(4.8) | 3(1.8) | 18(10.8) | 137(82.5) |  |  |
|  | **University/College** | **N** | 91(3.2) | 113(4.0) | 421(14.8) | 2214(78.0) |  |  | 39(1.4) | 53(1.9) | 269(9.5) | 2478(87.3) |  |  |
|  | **Master’s degree** | **N** | 4(2.5) | 7(4.5) | 23(14.6) | 123(78.3) |  |  | 1(0.6) | 1(0.6) | 18(11.5) | 137(87.3) |  |  |
|  | **PHD** | **N** | 2(8.3) | 1(4.2) | 2(8.3) | 19(79.2) |  |  | 0(0.0) | 1(4.2) | 1(4.2) | 22(91.7) |  |  |
| **Occupation** | **Health care worker** | **N** | 17(2.7) | 29(4.6) | 92(14.5) | 496(78.2) | **26.519** | **0.088** | 8(1.3) | 5(0.8) | 53(8.4) | 568(89.6) | **61.688** | <0.001 |
|  | **Government institution** | **N** | 7(2.5) | 10(3.5) | 27(9.5) | 239(84.5) |  |  | 4(1.4) | 2(0.7) | 12(4.2) | 265(93.6) |  |  |
|  | **Private institution** | **N** | 4(2.2) | 8(4.4) | 25(13.7) | 145(79.7) |  |  | 2(1.1) | 3(1.6) | 8(4.4) | 169(92.9) |  |  |
|  | **Business** | **N** | 12(6.1) | 9(4.5) | 36(18.2) | 141(71.2) |  |  | 7(3.5) | 8(4.0) | 32(16.2) | 151(76.3) |  |  |
|  |  | **%** | 6.1% | 4.5% | 18.2% | 71.2% |  |  | 3.5% | 4.0% | 16.2% | 76.3% |  |  |
|  | **Military** | **N** | 3(9.4) | 3(9.4) | 5(15.6) | 21(65.6) |  |  | 2(6.3) | 1(3.1) | 3(9.4) | 26(81.3) |  |  |
|  | **Unemployed** | **N** | 70(3.8) | 80(.4) | 281(15.4) | 1391(76.3) |  |  | 25(1.4) | 48(2.6) | 185(10.2) | 1564(85.8) |  |  |
|  | **Other** | **N** | 20(4.6) | 25(5.7) | 68(15.6) | 322(74.0) |  |  | 6(1.4) | 3(0.7) | 50(11.5) | 376(86.4) |  |  |
| **Economical status** | **Excellent** | **N** | 9(2.7) | 14(4.2) | 37(11.2) | 271(81.9) | **16.121** | **0.064** | 7(2.1) | 11(3.3) | 23(6.9) | 290(87.6) | **21.507** | **0.011*** |
|  | **Good** | **N** | 60(3.4) | 80(4.5) | 281(16.0) | 1340(76.1) |  |  | 20(1.1) | 35(2.0) | 189(10.7) | 1517(86.1) |  |  |
|  | **Moderate** | **N** | 47(3.8) | 55(4.4) | 185(14.8) | 960(77.0) |  |  | 18(1.4) | 18(1.4) | 111(8.9) | 1100(88.2) |  |  |
|  | **Poor** | **N** | 17(6.9) | 15(6.1) | 31(12.6) | 184(74.5) |  |  | 9(3.6) | 6(2.4) | 20(8.1) | 212(85.8) |  |  |
| **Housemate** | **0** | **N** | 3(6.5) | 2(4.3) | 6(13.0) | 35(76.1) | **41.699** | <0.001 | 1(2.2) | 0(0.0) | 7(15.2) | 38(82.6) | **19.706** | **0.003*** |
|  | **1-5** | **N** | 88(3.2) | 112(4.1) | 371(13.5) | 2180(79.2) |  |  | 35(1.3) | 50(1.8) | 238(8.7) | 2428(88.3) |  |  |
|  | **>5** | **N** | 42(5.3) | 50(6.3) | 157(19.9) | 540(68.4) |  |  | 18(2.3) | 20(2.5) | 98(12.4) | 653(82.8) |  |  |
